# Alcohol disrupts spatial coordination of autophagy–lysosome pathways to impair intestinal barrier integrity

**DOI:** 10.1016/j.ebiom.2026.106534

**Published:** 2026-10-26

**Authors:** Murugadas Anbazhagan, Ahmed Bakheet, John D. Dickinson, Ali Keshavarzian, Nghi V.N. Tran, Tin Nguyen, Paul G. Thomes

**Affiliations:** a Department of Anatomy, Physiology and Pharmacology, College of Veterinary Medicine, Auburn University, Auburn, AL, USA; b Department of Internal Medicine, University of Nebraska Medical Center, Omaha, NE, USA; c Rush Center for Integrated Microbiome and Chronobiology Research and Departments of Medicine, Physiology and Anatomy and Cell Biology, Rush University Medical Center, Chicago, IL, USA; d Department of Computer Science and Software Engineering, Auburn University, Auburn, AL, USA; e Department of Industrial and Systems Engineering, Wayne State University, Detroit, MI, USA; f Karmanos Cancer Institute, Wayne State University School of Medicine, Detroit, MI, USA

**Keywords:** Alcohol use disorder, Autophagy, Lysosomal remodelling, Claudin-2 trafficking, Epithelial stress, Gut barrier

## Abstract

**Background:**

Alcohol use disorder (AUD) drives intestinal barrier dysfunction, endotoxemia, and liver injury, yet the epithelial mechanisms remain undefined. Autophagy and lysosomes maintain intestinal epithelial homoeostasis and barrier integrity, but their response to chronic alcohol exposure is unclear.

**Methods:**

Human intestinal tissues from individuals with and without AUD were analysed alongside human intestinal organoids. Wild-type, CAG–RFP–EGFP–LC3, GFP–LC3B, and *Atg16l1* hypomorphic mice underwent chronic-binge ethanol feeding. Organoids and Caco-2 cells were exposed to ethanol (50 mM) with or without bafilomycin A1 or Torin-2. Autophagy–lysosome pathways were assessed using transcriptomics, immunofluorescence, biochemical fractionation, and flux reporters.

**Findings:**

Alcohol induced heterogeneous autophagy-lysosome responses characterised by lysosomal stress/adaptive remodelling. These changes occurred with limited autophagosome formation and maturation, resulting in impaired autophagic throughput. These responses differed along the crypt–villus axis and between intestinal regions. Disrupted autophagy was associated with altered trafficking and accumulation of claudin-2. *Atg16l1* hypomorphic mice exhibited increased baseline intestinal permeability vs wild-type controls (*p* = 0.03). Following ethanol exposure, *Atg16l1* hypomorphic mice showed greater intestinal permeability (*p* = 0.003), circulating and hepatic endotoxin (*p* = 0.002), hepatic triglycerides (*p* = 0.01), and serum ALT (*p* = 0.02) vs ethanol-fed wild-type mice.

**Interpretation:**

Alcohol disrupts intestinal autophagy–lysosome homoeostasis in a spatially heterogeneous manner, accompanied by defective epithelial proteostasis and tight junction dysregulation. Impaired autophagosome formation exacerbates gut permeability and liver injury, supporting intestinal autophagy as a potential therapeutic target in alcohol-associated disease. Broader applicability of the human findings requires validation in larger cohorts.

**Funding:**

This study was supported by grants from the National Institutes of Health.

**Research in context**

**Evidence before this study**

We searched PubMed for studies published up to March 2026 using combinations of the term's “alcohol”, “ethanol”, “intestinal epithelium”, “autophagy”, “lysosome”, and “intestinal barrier”. Alcohol-associated intestinal barrier dysfunction contributes to endotoxemia and liver injury. Autophagy–lysosome pathways maintain intestinal epithelial homoeostasis and regulate tight-junction proteins, and alcohol can alter autophagy in multiple organs. However, how alcohol affects autophagy–lysosome function within the intestinal epithelium, whether these effects vary by intestinal region and epithelial compartment, and whether alterations in this process contribute to barrier dysfunction and liver injury remain unclear.

**Added value of this study**

This study reveals that alcohol does not produce a uniform intestinal autophagy response but instead disrupts autophagy–lysosome coordination in region- and crypt-villus compartment-specific patterns. Integration of human tissues, organoids, and complementary mouse models links this spatial remodelling to altered claudin-2 trafficking. Importantly, genetic impairment of autophagy using *Atg16l1* hypomorphic mice demonstrates that reduced autophagy capacity worsens intestinal permeability, endotoxemia, and liver injury during alcohol exposure.

**Implications of all the available evidence**

Together, the evidence shifts the view of intestinal autophagy in alcohol-associated disease from a uniform pathway response to a spatially regulated determinant of epithelial barrier integrity. Intestinal region and epithelial compartment should therefore be considered when evaluating autophagy dysfunction and therapeutic strategies. Targeting autophagy-lysosome homoeostasis may offer an approach to preserve intestinal barrier function and limit downstream alcohol-associated liver injury.

## Introduction

Chronic heavy alcohol consumption disrupts cellular homoeostasis across multiple organ systems, producing pathological changes that extend beyond tissues directly exposed to alcohol.^1^, ^2^, ^3^, ^4^ While alcohol exerts direct cell-autonomous toxicity, increasing evidence indicates that the severity and progression of alcohol-associated disease are strongly influenced by dysregulated inter-organ communication.^5^, ^6^ Metabolic, inflammatory, and stress-responsive signals originating in one tissue can modify the function and vulnerability of distant organs, positioning alcohol-related pathology as a systemic disorder driven by altered biological signalling networks.^7^ In particular, gut-derived signals have emerged as key drivers of systemic inflammation and liver injury in alcohol-associated disease.

The intestine represents a critical early site of alcohol exposure and an upstream regulator of multi-organ injury.^8^, ^9^ Alcohol perturbs epithelial homoeostasis, alters microbial composition, and compromises barrier integrity, promoting the systemic dissemination of microbial and metabolic products that amplify inflammatory signalling in peripheral tissues.^10^, ^11^, ^12^ This gut barrier dysfunction is a major contributor to endotoxemia and the progression of alcohol-associated liver disease. Despite the recognised importance of the gut in alcohol-associated disease, the epithelial mechanisms that link alcohol exposure to barrier dysfunction and downstream organ injury remain incompletely understood.

Macroautophagy (hereafter autophagy) is a highly conserved lysosome-dependent degradative pathway essential for maintaining epithelial homoeostasis.^13^ Within the intestinal epithelium, autophagy supports diverse cell-type–specific functions that collectively preserve barrier integrity and host–microbe balance. In secretory lineages, autophagic machinery regulates mucin production and antimicrobial peptide processing, contributing to microbial containment at the mucosal surface.^14^, ^15^ In absorptive epithelial cells, autophagy and lysosomal pathways eliminate damaged organelles and intracellular pathogens, recycle macromolecules, and regulate tight-junction dynamics, thereby maintaining controlled permeability.^16^, ^17^, ^18^ Through these integrated actions, autophagy coordinates epithelial stress adaptation with immune balance and tissue resilience. Through these functions, autophagy directly contributes to epithelial barrier integrity and intestinal immune homoeostasis. Although alcohol has been reported to modulate autophagy in multiple organs,^19^ whether and how chronic alcohol exposure perturbs autophagy–lysosome organisation within the intestinal epithelium remains largely undefined.

Here, we investigated the impact of alcohol exposure on epithelial autophagy–lysosome regulation in the intestine. Using complementary analyses of human intestinal tissue, patient-derived organoids, and region- and epithelial compartment-resolved studies in the mouse intestine, we examined how alcohol alters epithelial autophagy–lysosome organisation and assessed the consequences of these changes for intestinal barrier integrity and epithelial homoeostasis. Our findings identify spatially organised disruption of autophagy–lysosome coordination as a key epithelial mechanism linking alcohol exposure to intestinal barrier dysfunction and gut–liver disease progression.

## Methods

### Ethics

#### Human studies

Formalin-fixed, paraffin-embedded (FFPE) intestinal tissue sections and human intestinal organoids generated from participant samples were obtained from Rush University Medical Centre under institutionally approved protocols. Participants provided informed consent for long-term storage and use of their samples under the Rush Centre for Integrated Microbiome and Chronobiology Research Data and Biorepository protocol (IRB #10062805). Fresh intestinal tissues were obtained from the Cooperative Human Tissue Network (CHTN) in accordance with CHTN policies and applicable ethical requirements. All materials were provided de-identified. Their use was reviewed by the Auburn University Institutional Review Board and determined to be exempt (IRB # STUDY00000518).

#### Animal studies

All animal procedures were conducted in accordance with applicable institutional guidelines and were approved by the Institutional Animal Care and Use Committees of Auburn University (IACUC # 2023–5353) and the University of Nebraska Medical Center (IACUC # 16-142-12-EP). Animal housing, husbandry, environmental enrichment, welfare monitoring, and humane endpoints were implemented in accordance with the respective institutionally approved IACUC protocols.

### Human intestinal tissue procurement

Human intestinal tissue was obtained from two independent sources. FFPE ileal and colonic tissue sections from female individuals with and without alcohol use disorder (AUD) were obtained through the R24 Alcohol Research Resource Program at Rush University Medical Center. AUD status was determined using the Alcohol Use Disorders Identification Test–Consumption (AUDIT-C).^20^ Relevant sample information is provided in Table S6. In addition, fresh human colonic tissue from individuals with no reported history of alcohol use and no histopathological abnormalities was obtained from CHTN, supported by the National Cancer Institute (NCI). Fresh tissues were derived from surgically resected specimens and included only samples deemed histologically normal by board-certified pathologists. Relevant sample information is provided in Table S7.

### Animal studies

Wild-type C57BL/6 (RRID: IMSR_JAX:000664) and CAG-RFP-EGFP-LC3 mice (RRID: IMSR_JAX:027,139; C57BL/6 background) were obtained from The Jackson Laboratory (Bar Harbour, ME). GFP-LC3B mice (BRC No. RBRC00806; C57BL/6 background) were obtained from RIKEN BRC, Japan. *Atg16l1* global hypomorphic (*Atg16l1*ˆhm/hm; MGI: 3818574) mice, which exhibit reduced *Atg16l1* expression on a C57BL/6 background were obtained from Dr. Thad Stappenbeck through the Mouse Genetics Project at the Wellcome Sanger Institute (PMID: 18849966). CAG-RFP-EGFP-LC3 and GFP-LC3B mice were bred and maintained under specific pathogen–free (SPF) conditions at the Auburn University animal care facility. These reporter lines enable complementary microscopic and biochemical assessment of autophagosomes, autolysosomes, and autophagy flux *in vivo*. *Atg16l1*ˆhm/hm mice were bred and maintained under SPF conditions at the University of Nebraska Medical Center animal care facility. Ten-week-old female mice were fed control or ethanol (EtOH) Lieber–DeCarli liquid diets using the chronic-binge EtOH feeding method in accordance with the established NIAAA model.^21^ For *Atg16l1*ˆhm/hm studies, age-matched male ATG16L1ˆhm/hm mice and their littermate wild-type controls were used. Mice were acclimated to a control liquid diet for 2 days, followed by weight matching and allocation to control and EtOH groups. EtOH-fed mice were pair-fed with corresponding control animals. The individual mouse was the experimental unit. EtOH-fed mice underwent a 3-day EtOH ramp (1–5%) and were maintained on a 5% EtOH diet for 10 days, while control mice received an isocaloric liquid diet. On day 10, EtOH-fed mice received a single EtOH gavage (5 g/kg body weight), and control mice received an isocaloric maltose dextrin gavage. Animals were euthanised 9 h post-gavage. A total of 42 mice (30 female and 12 male) were used: wild-type C57BL/6 mice (n = 8/group; 16 total), CAG-RFP-EGFP-LC3 reporter mice (n = 3/group; 6 total), GFP-LC3B reporter mice (n = 4/group; 8 total), and 12 mice in the *Atg16l1*ˆhm/hm experiment (*Atg16l1*ˆhm/hm and wild-type littermates; n = 3/group, four groups). As an additional assessment, sample sizes were evaluated using the resource-equation approach (E = total number of animals − total number of experimental groups).^22^ The principal wild-type experiment yielded E = 14, while the *Atg16l1*ˆhm/hm, RFP-GFP-LC3, and GFP-LC3 experiments yielded E = 8, 4, and 6, respectively. Exact sample sizes for individual analyses are provided in the corresponding figure legends.

### Human intestinal organoid culture and ethanol exposure

Human colonic organoid cultures were established as previously described.^23^ Briefly, human colonic tissues were incubated in 15 mmol/L EDTA for 45 min, after which epithelial crypts were isolated by microdissection under a stereomicroscope. Isolated crypts were washed and embedded in Matrigel (Corning, cat. no. 354230) and cultured in Intesticult™ Human Organoid Growth Medium (STEMCELL Technologies, cat. no. 100–0190). After 5–7 days, mature organoids were either cryopreserved or passaged at equal density, and organoids between passages 3 and 5 were subsequently exposed to ethanol (50 mM) for 24 h prior to downstream analyses.

### Caco2 cell line experiments

Caco-2 cells were obtained from the American Type Culture Collection (ATCC) RRID: CVCL_0025 and maintained in high-glucose DMEM supplemented with 10% foetal bovine serum, 100 U/ml penicillin, and 100 μg/ml streptomycin. All experiments were performed on fully differentiated cells. Cells were treated with the indicated agents in the presence or absence of ethanol (50 mM, 24 h).^24^, ^25^, ^26^ Where indicated, cells were co-treated with bafilomycin A1 (Sigma–Aldrich, cat.no. 88899-55-2), chloroquine (Sigma–Aldrich cat.no. C6628), or Torin2 (Sigma–Aldrich 1223001-51-1).

#### Statistical analysis

Sample sizes were based on prior experience with the chronic-binge ethanol feeding model, animal and tissue availability, and the requirements of individual experimental endpoints; no formal a priori sample-size calculation was performed. Data are presented as mean ± SEM. Normality was assessed for the principal datasets using the Shapiro–Wilk test, for both the wild-type and *Atg16l1*ˆhm/hm datasets and no significant departures from normality were detected. Datasets with small group sizes (n = 3–4/group) were also examined for distributional irregularities. Statistical significance between two independent groups was determined using a two-tailed, unpaired student's *t*-test. Three outlier data points across wild-type mouse Western blot analyses were excluded using Grubbs' test; no animals were excluded. Experiments involving multiple groups were analysed by two-way ANOVA followed by Tukey's post hoc multiple-comparisons test. Statistical analyses were performed using GraphPad Prism software.

#### Role of funders

The funders had no role in study design, data collection, data analysis, data interpretation, or writing of the report.

## Results

### Alcohol use disorder is associated with lysosomal remodelling of the autophagy-lysosome system in the human intestine

To determine whether alcohol use disorder is associated with remodelling of autophagy–lysosome pathways in the human intestine, a key tissue implicated in alcohol-associated barrier dysfunction, we reanalysed a previously published bulk RNA-seq dataset from duodenal biopsies of individuals with and without AUD. Of ∼15,000 detected transcripts, 423 (∼2.8%) were autophagy–lysosome related and included for pathway-focused analysis (Tables S1–S5). Among these, 121 transcripts were differentially expressed in AUD compared with controls (101 increased, 20 decreased; Fig. 1a–e). Autophagosome, lysosomal, and autophagosome–lysosome interface categories exhibited coordinated remodelling. Autophagosome-associated transcripts showed increased maturation-related genes (e.g., *GABARAP, ATG101, ATG4A/ATG4B*) with reduced initiation-related transcripts (e.g., *ATG9B, ULK2*). Lysosome-related transcripts demonstrated increased biogenesis and acidification components (e.g., *LAMP1, HSPA8, ATP6V1D, ATP6V0E1*) alongside reduced hydrolase transcripts (e.g., *CTSB, CTSC*). The interface category showed the greatest remodelling (57/164 altered), including increased endosomal sorting and membrane trafficking components (e.g., *CHMP2A, CHMP3, VPS52, YKT6*) and reduced negative regulators of autophagosome biogenesis (*TBC1D5, TBC1D14*). Regulatory transcripts were also altered (20/99), including increased stress-sensing genes such as *MTOR*.

**Fig. 1:**
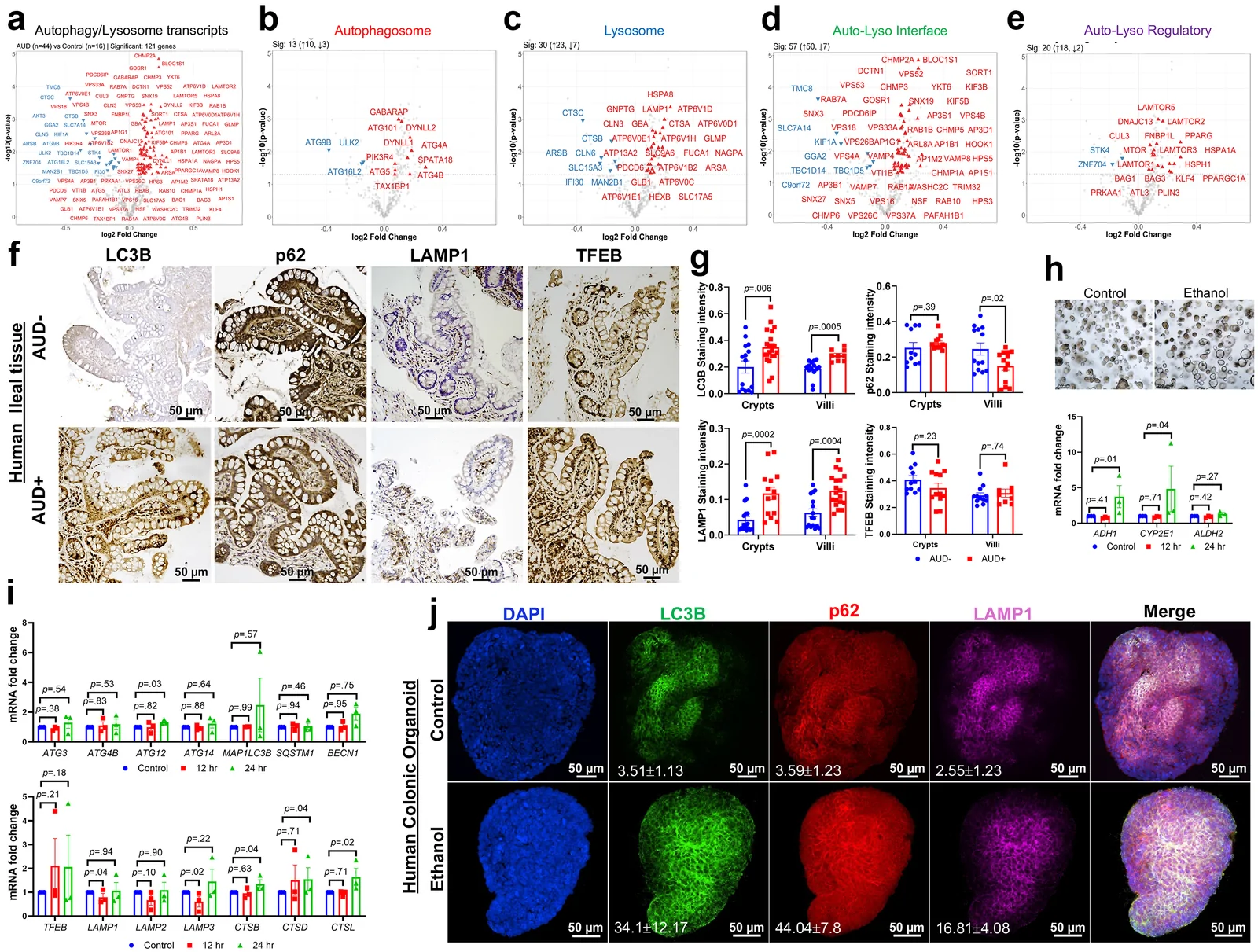
**Alcohol use disorder is associated with autophagy–lysosome remodelling across human intestinal tissues and organoids.** Volcano plots showing (**a**) all differentially expressed transcripts, (**b**) autophagosome-related transcripts, (**c**) lysosome-related transcripts, (**d**) autophagosome–lysosome interface–related transcripts, and (**e**) autophagosome–lysosome regulatory transcripts identified by bulk RNA-seq analysis of duodenal biopsies from individuals with AUD (AUD+; *n* = 44) and without AUD (AUD−; *n* = 16). Upregulated transcripts are shown in red and downregulated transcripts in blue. (**f**) Representative immunohistochemical images and (**g**) quantification of staining intensities of LC3B, p62, LAMP1, and TFEB in crypt and villus compartments of human ileal sections from individuals with AUD (AUD+) and without AUD (AUD−) (*n* = 3 per group). For quantification, a minimum of 3–5 crypts and villi per sample were analysed, yielding a total of at least 9–15 crypts and villi per group across three independent donor samples. (**h**) Representative bright-field micrographs and mRNA expression of ethanol-metabolising genes, and mRNA levels of (**i**) autophagosome- and lysosome-related genes in normal human colonic organoids treated with and without 50 mM ethanol for 12 and 24 h (*n* = 3 independent donor-derived organoids). (**j**) Representative immunofluorescence images and puncta quantification of LC3B (green), p62 (red), and LAMP1 (magenta) in normal human colonic organoids exposed to 0 or 50 mM ethanol for 24 h. Puncta quantification is shown at the bottom left of the micrographs. Quantification was performed using three organoids per donor from three independent donor-derived organoid lines (total of nine organoids per group), with each organoid containing approximately 500 cells, yielding ∼4500 cells per group for analysis. Statistical comparisons between two groups were performed using two-tailed, unpaired student's *t*-tests; RNA-seq data were analysed as described in the Methods.

To evaluate protein-level changes *in situ*, we immunostained human ileal tissues for autophagy–lysosome markers. Immunohistochemistry analysis revealed increased LC3B and LAMP1 staining in individuals with AUD across both crypt and villus epithelial compartments of the ileum. LC3B and LAMP1 staining increased by approximately 1.7-fold (*p* = 0.006) and 2.7-fold (*p* = 0.0002) in crypts and by ∼1.5-fold (*p* = 0.0005) and 2.0-fold (*p* = 0.0004) in villi, respectively. TFEB expression remained unchanged as no significant differences were detected in total (Fig. 1g), nuclear and cytoplasmic TFEB (Fig. S1) staining intensities. The autophagy adaptor p62 remained unchanged in crypts (*p* = 0.39) but decreased by ∼1.6-fold (*p* = 0.02) in villi (Fig. 1f and g).

To determine whether autophagy–lysosome alterations observed in human ileal tissue are recapitulated in donor-derived epithelial systems, we next assessed mRNA expression of autophagosome-related genes (*ATG3, ATG4B, ATG12, ATG14, SQSTM1*, and *BECN1*) and lysosome-associated genes (*TFEB, LAMP1, LAMP2, LAMP3, CTSB, CTSD, CTSL, ATP6V1D, ATP6V1H*, and *ATP6V0D2*) in colonic organoids derived from individuals with and without AUD. Transcript abundance did not differ between groups (Fig. S2a–c).

We next transduced organoids with a tandem RFP–GFP–LC3B reporter and exposed them to 0 or 50 mM ethanol for 24 h to assess compartment acidification.^27^ GFP-only, yellow (GFP + RFP+), and red (RFP-only) puncta were interpreted as early, non-acidified, and acidified compartments, respectively. Under basal conditions, organoids derived from individuals with AUD showed unchanged GFP-only puncta (*p* > 0.9999) but reduced yellow (*p* = 0.02) and red puncta (*p* = 0.004) compared with those derived from individuals without AUD. Consistent with this, the red fraction, representing the proportion of acidified puncta (red-only) relative to total LC3B puncta (yellow + red), was reduced in organoids derived from individuals with AUD (*p* = 0.04). In organoids derived from individuals without AUD, ethanol exposure did not alter GFP-only (*p* = 0.58) or yellow puncta (*p* = 0.58) but reduced red puncta (*p* = 0.04) and the red fraction (*p* = 0.04). In contrast, ethanol-exposed organoids derived from individuals with AUD showed unchanged puncta counts, while the red fraction increased (*p* = 0.04) (Fig. S3a–m).

To determine whether ethanol exposure alone induces autophagy–lysosome remodelling independent of AUD origin, we examined ethanol responses in colonic organoids derived from individuals with no reported history of alcohol use. We assessed epithelial cell–intrinsic transcriptional responses and marker localisation and first confirmed ethanol responsiveness by measuring alcohol-metabolising enzymes after 12 and 24 h exposure to 50 mM ethanol. *ADH1* (*p* = 0.01) and *CYP2E1* (*p* = 0.04) mRNA levels increased at 24 h, whereas ALDH2 expression remained unchanged (*p* = 0.27) (Fig. 1h).

We next examined whether ethanol alters autophagy–lysosome transcriptional programs in human colonic organoids. After 12 and 24 h exposure to 50 mM ethanol, autophagosome-related transcripts (*ATG3, ATG4B, ATG12, ATG14, MAP1LC3B*, *SQSTM1*, and *BECN1*) showed modest upward trends at 24 h, with *ATG12* emerging as the only significantly induced gene (*p* = 0.03) (Fig. 1i). In contrast, lysosome-associated transcripts displayed a time-dependent response. TFEB trended upward without significance, whereas lysosomal membrane components were transiently reduced (*LAMP1* (*p* = 0.04) and *LAMP3* (*p* = 0.02) decreased at 12 h and returned to baseline by 24 h; *LAMP2* unchanged). Notably, lysosomal protease transcripts (*CTSB* (*p* = 0.04)*, CTSD* (*p* = 0.04)*, and CTSL* (*p* = 0.02)) were significantly increased at 24 h, consistent with preferential activation of lysosomal degradative capacity (Fig. 1i).

Consistent with these transcriptional changes, immunofluorescence analyses of colonic organoids showed that ethanol exposure increased LC3B, p62, and LAMP1 puncta by approximately 9.7-fold (*p* = 0.04), 12.2-fold (*p* = 0.006), and 6.6-fold (*p* = 0.01) fold, respectively (Fig. 1j). Colocalisation analysis showed increased LC3B–p62 association following ethanol exposure (*p* = 0.0001), along with a modest but significant increase in p62–LAMP1 colocalisation (*p* = 0.008), whereas LC3B–LAMP1 colocalisation (*p* = 0.10) exhibited only a nonsignificant upward trend (Fig. S4). Statistical comparisons between two groups were performed using two-tailed, unpaired student's *t*-tests; RNA-seq data were analysed as described in the methods.

### Ethanol feeding elicits divergent autophagy-lysosome responses in villus and crypt compartments of the mouse duodenum

To determine whether autophagy–lysosome alterations observed in the human intestine are conserved *in vivo*, we examined ethanol-induced responses in the mouse duodenum across crypt and villus epithelial compartments. Immunofluorescence showed ∼1.5-fold (*p* = 0.03), 2.5-fold (*p* = 0.01), and 1.7-fold (*p* = 0.01) fold increases in LC3B, p62, and LAMP1 puncta numbers, respectively, in villus epithelial cells of ethanol-fed mice (Fig. 2a). Within the epithelial cells, LC3B and LAMP1 localised predominantly to the apical region near tight junctions, whereas p62 was enriched basally (Fig. S5a). Ethanol feeding increased p62 (*p* = 0.01) and LAMP (*p* = 0.0007) positive puncta size without altering LC3B puncta size (*p* = 0.052) (Fig. S5c–e).

**Fig. 2:**
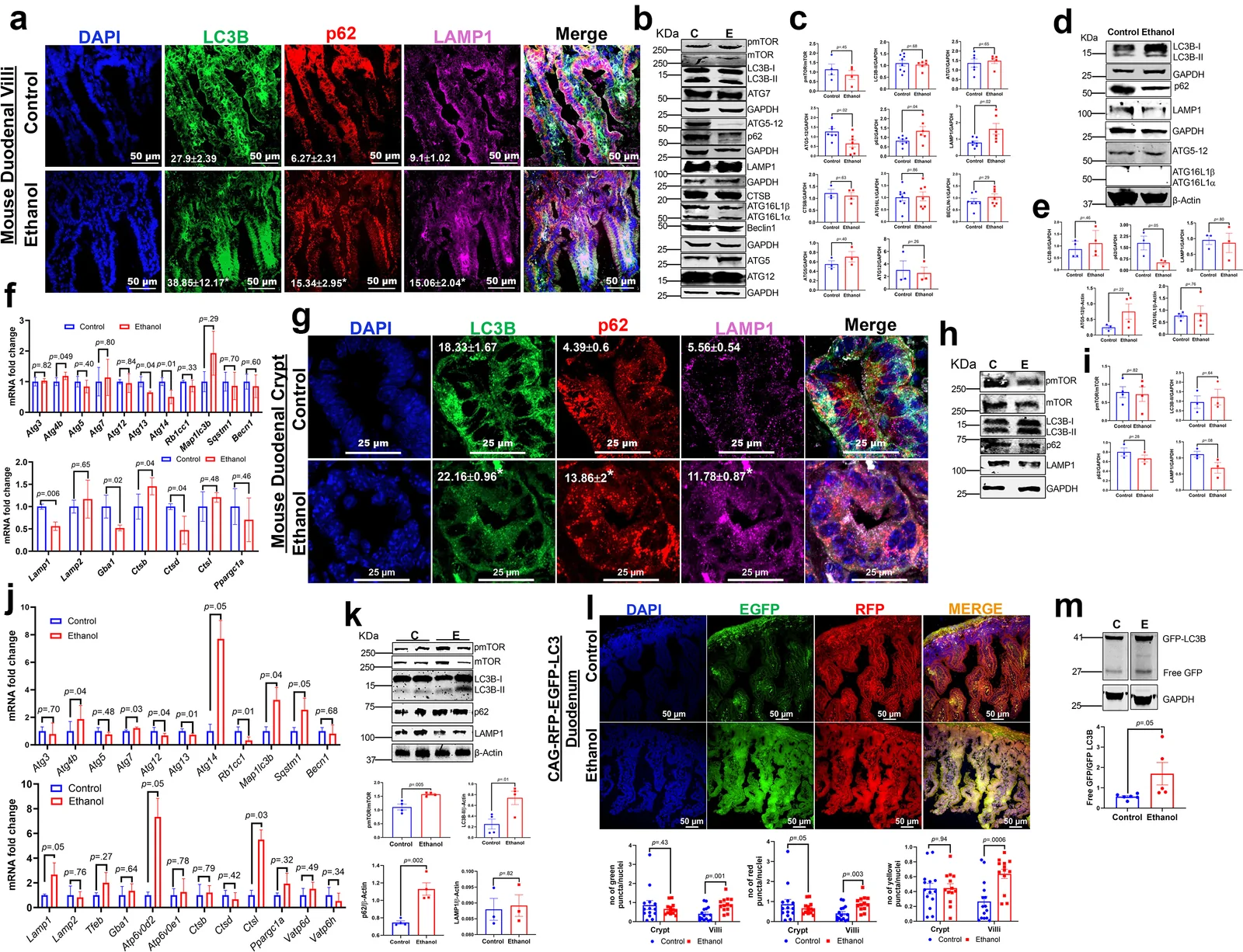
**Ethanol feeding differentially regulates autophagy–lysosome dynamics across duodenal epithelial compartments.** (**a**) Representative immunofluorescence images of the duodenal villus compartment from control- and ethanol diet–fed mice stained for LC3B (green), p62 (red), and LAMP1 (magenta) (*n* = 4 animals per group). Puncta quantification is shown at the bottom left of the micrographs. (**b, c**) Representative immunoblots and densitometric quantification of autophagy- and lysosome-related proteins in soluble mucosal fractions (*n* = 6–8 animals per group) and (**d, e**) insoluble mucosal fractions (*n* = 3–4 animals per group) isolated from the duodenum of control- and ethanol-fed mice. (**f**) mRNA expression levels of autophagy-related and lysosome-related transcripts, respectively, in isolated duodenal crypts from control- and ethanol-fed mice (*n* = 4 animals per group). (**g**) Representative immunofluorescence images of the duodenal crypt compartment from control- and ethanol diet–fed mice stained for LC3B (green), p62 (red), and LAMP1 (magenta) (*n* = 4 animals per group). Puncta quantification is shown at the bottom left of the micrographs. (**h**) Representative immunoblot images and (**i**) densitometric quantification of autophagy–lysosome markers in crypt lysates (*n* = 3 animals per group) (**j**) mRNA expression levels of autophagy-related and lysosome-related transcripts, respectively, in whole duodenal mucosa (*n* = 4–8 animals per group), and (**k**) representative immunoblots and densitometric quantification of the indicated autophagy–lysosome markers in duodenal mucosal homogenates from control- and ethanol-fed mice (*n* = 4 animals per group). (**l**) Representative immunofluorescence images and quantification of EGFP^+^RFP^−^ (green) puncta, EGFP^+^RFP^+^ (yellow) puncta, and EGFP^−^RFP^+^ (red) puncta in crypt and villus compartments of duodenal sections from control- and ethanol-fed CAG-RFP-EGFP-LC3B reporter mice (*n* = 3 animals per group). For immunofluorescence analyses, a minimum of 5 crypts and 3 villi per mouse were quantified across 3 mice per group, yielding a total of 15 crypts and 9 villi per group. (**m**) Representative immunoblots and densitometric quantification of free GFP and conjugated GFP–LC3B (LC3-I) in soluble fractions from the duodenum of control- and ethanol-fed GFP–LC3B mice (*n* = 4–6 animals per group). Free GFP was normalised to conjugated GFP–LC3B-I, with GAPDH as a loading control. Non-adjacent control and ethanol-treated lanes from the same membrane were assembled for presentation, with a dividing line indicating the splice. Statistical comparisons between two groups were performed using two-tailed, unpaired student's *t*-tests.

In line with this spatial remodelling, soluble fractions of duodenal mucosal scrapings from ethanol-fed mice exhibited approximately 1.6-fold (*p* = 0.04) and 3.1-fold (*p* = 0.02) increases in p62 and LAMP1 protein levels, respectively, accompanied by a ∼1.8-fold (*p* = 0.02) decrease in the ATG12–ATG5 conjugate, a complex essential for autophagosome formation and maturation (Fig. 2b and c). In contrast, protein levels of LC3B-II, ATG7, ATG16L1, BECLIN-1, and the free forms of ATG5 and ATG12 were unchanged. In insoluble fractions, p62 levels were reduced by ∼3.5-fold (*p* = 0.05), whereas LC3B-II (*p* = 0.46), LAMP1(*p* = 0.80), ATG5–ATG12 conjugate (*p* = 0.22), and total ATG16L1 (*p* = 0.76) levels remained unchanged (Fig. 2d and e).

To determine whether ethanol-induced autophagy–lysosome remodelling extended to the crypt epithelium, we examined transcriptional changes in isolated crypts. qPCR analyses revealed selective alterations in autophagy- and lysosome-related transcripts following ethanol exposure. Among autophagosome-associated transcripts (*Atg3, Atg4b, Atg5, Atg7, Atg12, Atg13, Atg14, Rb1cc1, Map1lc3b, Sqstm1,* and *Becn1*), *Atg4b* mRNA increased, whereas *Atg13* and *Atg14* decreased. The expressions of the remaining transcripts were unchanged. Among lysosome-related transcripts (*Lamp1, Lamp2, Gba1, Ctsb, Ctsd, Ctsl,* and *Ppargc1a*) *Lamp1* and *Ctsd* decreased and *Ctsb* increased, with no other significant changes (Fig. 2f).

To determine whether these transcriptional changes were reflected at the cellular level, we examined marker distribution by immunofluorescence. Similar to the villus compartment, LC3B, p62, and LAMP1 puncta numbers increased by ∼1.2-, 3.1-, and 2.1-fold, respectively, in crypt epithelial cells of ethanol-fed mice (Fig. 2g). Ethanol feeding also increased p62-(*p* = 0.001) and LAMP1-(*p* = 0.001) positive puncta size without altering LC3B puncta size (*p* = 0.07) (Fig. S5b–e). Biochemical analysis of crypt lysates showed no significant differences in the phosphorylated mTOR–to–total mTOR ratio (*p* = 0.82) or in LC3B-II (*p* = 0.64), p62 (*p* = 0.28), and LAMP1 (*p* = 0.08) protein levels (Fig. 2h and i).

To assess whether these epithelial compartment-specific alterations were reflected at the tissue level, we next examined transcriptional and protein expression in total duodenal mucosa. Transcript analysis showed increased *Atg4b, Atg7, Atg14, Map1lc3b, Sqstm1, Lamp1, Atp6v0d2*, and *Ctsl* and decreased *Atg12, Atg13, and Rb1cc1* in ethanol-fed mice, whereas *Atg3, Atg5, Becn1, Lamp2, Tfeb, Gba1, Atp6v0e1, Ctsb, Ctsd, Ppargc1a, Atp6v1d*, and *Atp6v1h* were unchanged (Fig. 2j). In whole duodenal mucosal homogenates, the phosphorylated mTOR–to–total mTOR ratio increased ∼2-fold (*p* = 0.005), accompanied by ∼3-fold (*p* = 0.01) and ∼2- fold (*p* = 0.002) increases in LC3B-II and p62, respectively, while LAMP1 (*p* = 0.82) levels were unchanged (Fig. 2k).

To further assess autophagy dynamics across epithelial compartments, autophagic flux was analysed in duodenal tissue from CAG-RFP-EGFP-LC3B reporter mice, to assess the acidification status of LC3B-positive compartments. Ethanol feeding did not alter yellow (*p* = 0.94), or green puncta (*p* = 0.43), in crypt epithelial cells but significantly increased both populations in villi. In contrast, red puncta decreased in crypts (*p* = 0.05) and increased (*p* = 0.003) in villi, consistent with compartment-specific differences in LC3-positive compartment acidification along the duodenal crypt–villus axis (Fig. 2l). Autophagy activity was also assessed by measuring free GFP in the soluble fraction of mucosal scrappings from GFP-LC3B reporter mice. Free GFP levels did not increase significantly following ethanol feeding (Fig. 2m). Statistical comparisons between two groups were performed using two-tailed, unpaired student's *t*-tests.

### Ethanol feeding alters autophagy-lysosome regulation in the mouse ileum

Given the compartment-specific autophagy–lysosome alterations observed in the duodenum, we next examined whether similar patterns occur in the ileum. Immunofluorescence revealed increased puncta numbers of LC3B, p62, and LAMP1 in ileal villi of ethanol-fed mice, with approximately 5.6-fold, 2.8-fold, and 2.4-fold increases, respectively (Fig. 3a). Puncta size for each marker remained unchanged (Fig. S6a, c–e, f).

**Fig. 3:**
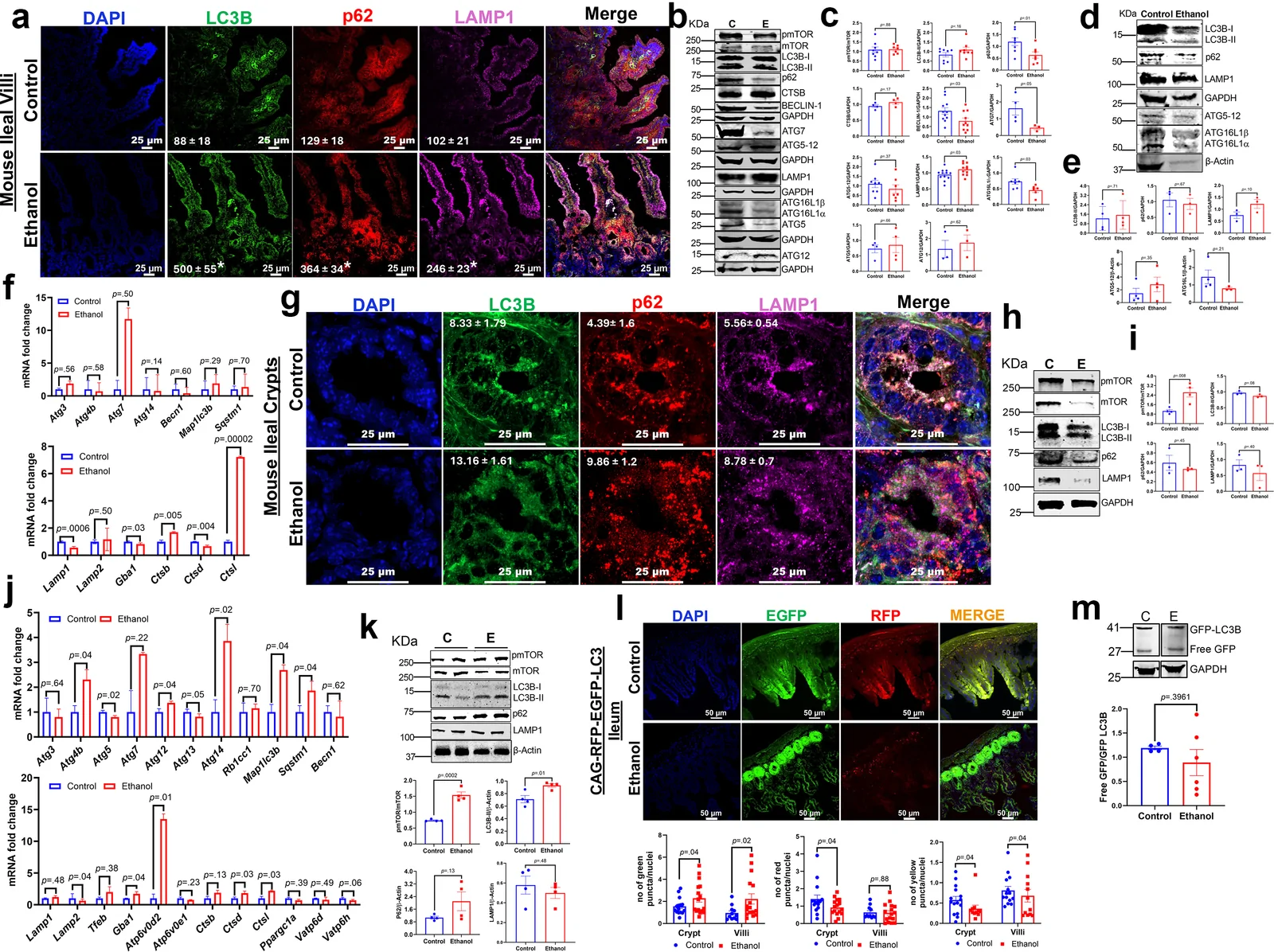
**Ethanol feeding differentially regulates autophagy dynamics along the ileal crypt–villus axis in mice.** (**a**) Representative immunofluorescence images of the ileal villus compartment from control- and ethanol diet–fed mice stained for LC3B (green), p62 (red), and LAMP1 (magenta). Puncta quantification is shown at the bottom left of the micrographs (*n* = 4–6 animals per group). (**b, c**) Representative immunoblots and densitometric quantification of autophagy- and lysosome-related proteins in soluble mucosal fractions (*n* = 4–8 animals per group) and (**d, e**) insoluble mucosal fractions (*n* = 3–4 animals per group) isolated from the ileum of control- and ethanol-fed mice. (**f**) mRNA expression levels of autophagy-related and lysosome-related transcripts, respectively, in isolated ileal crypts from control- and ethanol-fed mice (*n* = 3–4 animals per group). (**g**) Representative immunofluorescence images of the ileal crypt compartment from control- and ethanol diet–fed mice stained for LC3B (green), p62 (red), and LAMP1 (magenta). Puncta quantification is shown at the top left of the micrographs (*n* = 4 animals per group) (**h**) Representative immunoblot images and (**i**) densitometric quantification of autophagy–lysosome markers in crypt lysates (*n* = 3–4 animals per group) (**j**) mRNA expression levels of autophagy-related and lysosome-related transcripts, respectively, in whole ileal mucosa (*n* = 4–6 animals per group), and (**k**) representative immunoblots and densitometric quantification of the indicated autophagy–lysosome markers in total ileal mucosa from control- and ethanol-fed mice (*n* = 3–4 animals per group). (**l**) Representative immunofluorescence images and quantification of EGFP^+^RFP^−^ (green) puncta, EGFP^+^RFP^+^ (yellow) puncta, and EGFP^−^RFP^+^ (red) puncta, in crypt and villus compartments of ileal sections from control- and ethanol-fed CAG-RFP-EGFP-LC3B reporter mice (*n* = 3 animals per group). For flux analyses, a minimum of 5 crypts and 3 villi per mouse were quantified across 3 mice per group, yielding a total of 15 crypts and 9 villi per group. (**m**) Representative immunoblots and densitometric quantification of free GFP and conjugated GFP–LC3B (LC3-I) in soluble fractions from the ileum of control- and ethanol-fed GFP–LC3B mice (*n* = 4–6 animals per group). Free GFP was normalised to conjugated GFP–LC3B-I, with GAPDH shown as a loading control. Non-adjacent control and ethanol-treated lanes from the same membrane were assembled for presentation, with a dividing line indicating the splice. Quantification was performed as described for the duodenum (Fig. 2m). Statistical comparisons between two groups were performed using two-tailed, unpaired student's *t*-tests.

To determine whether these microscopic changes were accompanied by biochemical alterations in autophagy signalling, we analysed soluble fractions of ileal mucosal scrapings. In ethanol-fed mice, protein levels of ATG7, total ATG16L1, and BECLIN-1 decreased by 4-fold (*p* = 0.05), 1.6-fold (*p* = 0.03), and 2-fold (*p* = 0.03), respectively (Fig. 3b and c). The autophagy adaptor p62 decreased by 2-fold (*p* = 0.01), whereas LAMP1 increased by 1.3-fold (*p* = 0.03). In contrast, LC3B-II levels (*p* = 0.16), the phosphorylated mTOR–to–total mTOR ratio (*p* = 0.88), the ATG5–ATG12 conjugate (*p* = 0.37), cathepsin B (CTSB) (*p* = 0.17), and the free forms of ATG5 (*p* = 0.66) and ATG12 (*p* = 0.62) were unchanged. Insoluble fractions showed no significant changes in LC3B-II (*p* = 0.71), p62 (*p* = 0.67), LAMP1 (*p* = 0.10), ATG5–ATG12 conjugate (*p* = 0.35), or total ATG16L1 (*p* = 0.21) (Fig. 3d and e).

To determine whether microscopic and biochemical changes in the crypt epithelium were linked to transcriptional regulation of autophagy–lysosome machinery, we analysed autophagy- and lysosome-related mRNAs in isolated ileal crypts. Among autophagosome-associated transcripts, *Atg3*, *Atg7*, *Map1lc3b*, and *Sqstm1* showed modest upward trends, whereas *Atg4b*, *Atg14*, and *Becn1* showed modest downward trends in ethanol-fed mice; however, none reached statistical significance. Among lysosome-associated transcripts (*Lamp1*, *Lamp2*, *Gba1*, *Ctsb*, *Ctsd*, and *Ctsl*), *Gba1* and *Ctsd* decreased and *Ctsb* and *Ctsl* increased, whereas *Lamp1* and *Lamp2* remained unchanged (Fig. 3f).

Similar to the villus compartment, LC3B, p62, and LAMP1 puncta numbers increased by approximately 1.6-fold (*p* = 0.04), 2.3-fold (*p* = 0.007), and 1.6-fold (*p* = 0.03), respectively, in ileal crypts of ethanol-fed mice (Fig. 3g), without changes in puncta size (Fig. S6g–j). In contrast, biochemical analysis of isolated ileal crypt fractions showed no significant differences in p62 (*p* = 0.45), or LAMP1 (*p* = 0.40), protein levels between control and ethanol-fed mice. Notably, the phosphorylated mTOR–to–total mTOR ratio increased by ∼3-fold (*p* = 0.008), whereas LC3B protein levels were reduced (*p* = 0.08) (Fig. 3h and i).

To determine whether these compartment-level alterations extended to the tissue level, we analysed total ileal mucosa. Transcript analysis showed significant increases in autophagy-related mRNAs (*Atg4b*, *Atg12*, *Atg14*, *Map1lc3b*, and *Sqstm1*) and reductions in *Atg5* and *Atg13* in ethanol-fed mice, whereas *Atg3*, *Atg7*, *Rb1cc1*, and *Becn1* remained unchanged. Among lysosome-associated transcripts (*Lamp1*, *Lamp2*, *Tfeb*, *Gba1*, *Atp6v0d2*, *Atp6v0e1*, *Ctsb*, *Ctsd*, *Ctsl*, *Ppargc1a*, *Atp6v1d*, and *Atp6v1h*), *Lamp2* decreased, whereas *Gba1*, *Atp6v0d2*, *Ctsd*, and *Ctsl* increased; others were unchanged (Fig. 3j). Analysis of total ileal mucosal homogenates showed an approximately 2-fold (*p* = 0.0002) increase in the phosphorylated mTOR–to–total mTOR ratio and increased LC3B-II (*p* = 0.01), protein levels, whereas p62 (*p* = 0.13), and LAMP1 (*p* = 0.48), remained unchanged (Fig. 3k).

To further define epithelial compartment-specific autophagy dynamics in the ileum, autophagic flux was analysed in CAG-RFP-EGFP-LC3B reporter mice, with LC3B puncta classified as described above. Ethanol feeding decreased GFP + RFP+ (yellow) puncta in both crypt (*p* = 0.04) and villi (*p* = 0.04) epithelial cells. GFP+ (green) puncta increased in both compartments, whereas GFP−RFP+ (red) puncta decreased in crypts (*p* = 0.04), and remained unchanged (*p* = 0.88), in villi (Fig. 3l). Autophagy activity was also assessed by measuring free GFP in soluble fraction of mucosal scrappings from GFP-LC3B reporter mice. Free GFP levels were lower following ethanol feeding but did not differ significantly from controls (*p* = 0.39) (Fig. 3m). Statistical comparisons between two groups were performed using two-tailed, unpaired student's *t*-tests.

### Claudin-2 spatially associates with autophagy-lysosome machinery across human and mouse intestinal epithelium

Because autophagy–lysosome markers localise apically near tight junctions (Fig. S4a, f), we examined whether the tight junction protein claudin-2, a pore-forming protein implicated in epithelial stress and barrier remodelling,^12^ spatially associates with autophagy–lysosome machinery in the intestinal epithelium. Since tight junctions along the villus epithelium represent the first epithelial structures exposed to luminal contents, analyses focused primarily on the villus compartment.^28^ Crypt analyses are presented in Fig. S7 and summarised below.

Total claudin-2 staining intensity was significantly reduced in ileal epithelium from individuals with AUD compared with individuals without AUD (*p* = 0.05) (Fig. 4a–c). Manders' colocalisation analysis demonstrated that claudin-2 association with LC3B and LAMP1 was comparable between the two groups, with both claudin-2–LC3B and claudin-2–LAMP1 overlap coefficients remaining approximately 0.5 (Fig. 4d and e). Within ileal epithelium from individuals without AUD, claudin-2 showed similar spatial association with LC3B and LAMP1. A comparable pattern was observed in samples from individuals with AUD, where claudin-2 association with LC3B and LAMP1 also remained similar based on Manders’ coefficients (Fig. 4f).

**Fig. 4:**
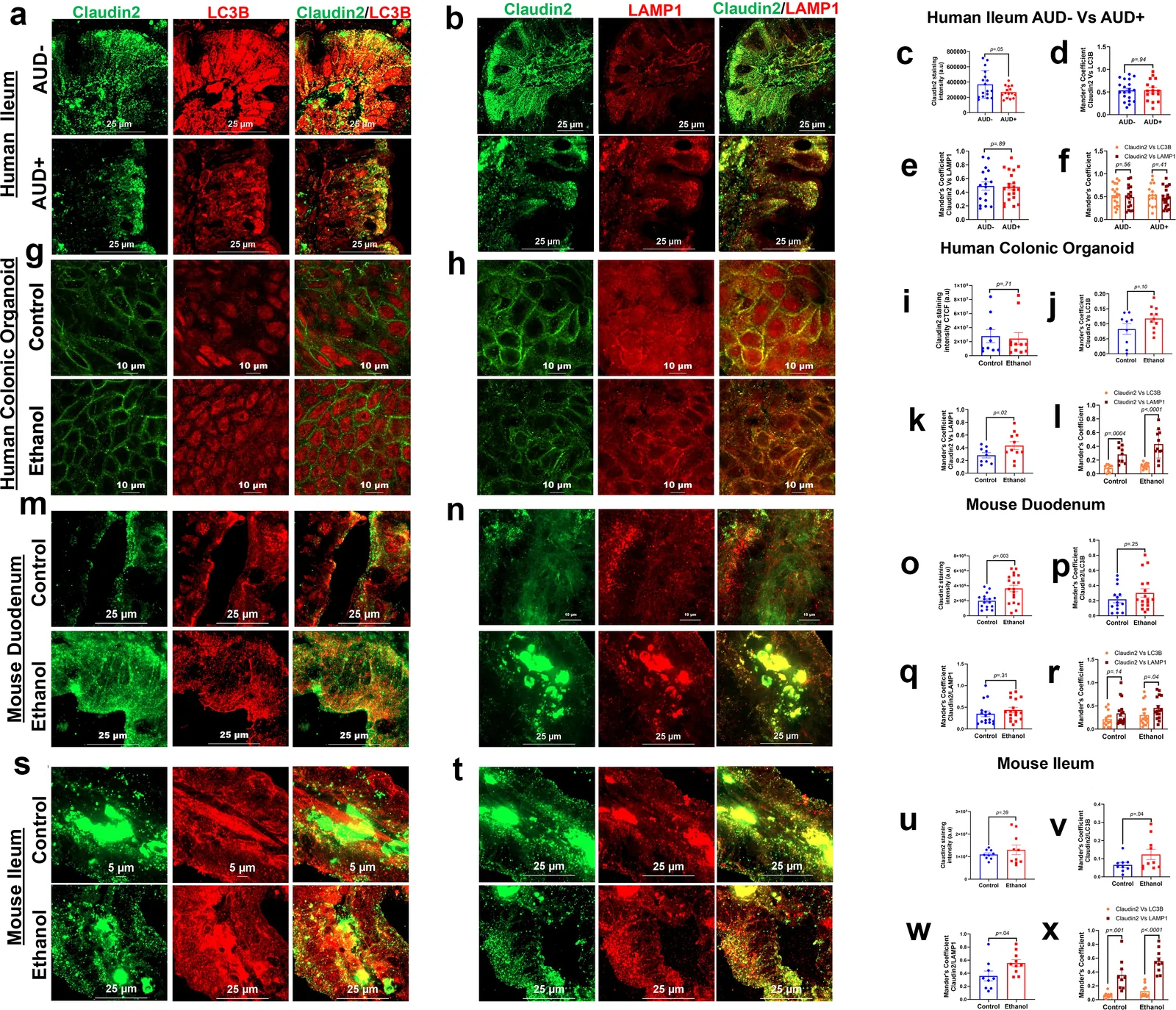
**Ethanol exposure alters claudin-2 association with autophagy–lysosome compartments across human and mouse intestinal epithelium.** Representative immunofluorescence micrographs depicting colocalisation of claudin-2 with LC3B and LAMP1, together with corresponding quantification of staining intensity and Manders' colocalisation coefficients, are shown for each experimental condition as indicated. (**a–f**) Ileal villi from individuals with AUD (AUD+) and without AUD (AUD−) (*n* = 3 per group). (**g–l**). Normal human colonic organoids exposed to 50 mM ethanol for 24 h (*n* = 3 independent donor-derived organoids). (**m–r**) Duodenal villi from control- and ethanol-fed mice (*n* = 4 animals per group). (**s–x**) Ileal villi from control- and ethanol-fed mice (*n* = 4 animals per group). For human ileal tissue, 3–6 villi per donor were quantified across three independent donors, yielding 9–18 villi per group. For organoid analyses, quantification was performed using 3 or 4 organoids per donor from three independent donor-derived organoid lines (total 9 or 12 organoids per group), with each organoid containing approximately 500 cells, yielding ∼4500 cells per group for analysis. For mouse studies, a minimum of 3 villi per mouse were quantified across four mice per group (total 9 villi per group). Statistical comparisons between two groups were performed using two-tailed, unpaired student's *t*-tests.

In human colonic organoids, total claudin-2 staining intensity remained unchanged (*p* = 0.71) following 24 h exposure to 50 mM ethanol compared with controls (Fig. 4g–i). Claudin-2–LC3B colocalisation was low and unchanged (*p* = 0.10) (Manders’ coefficient ∼0.06 in both conditions) (Fig. 4j). In contrast, claudin-2–LAMP1 colocalisation increased significantly (*p* = 0.02) following ethanol exposure (Fig. 4k). In both control and ethanol-treated organoids, claudin-2 association with LAMP1 was significantly greater than with LC3B (control, *p* = 0.0004; ethanol, *p* < 0.0001; Fig. 4l).

We next examined claudin-2 localisation in mouse duodenum and ileum. Duodenal claudin-2 staining intensity increased significantly in ethanol-fed mice compared with controls (*p* = 0.003) (Fig. 4m–o). However, Manders’ coefficients for claudin-2 colocalisation with LC3B (*p* = 0.25) or LAMP1 (*p* = 0.31) were unchanged between control and ethanol-fed groups (Fig. 4p and q). Within both control and ethanol-fed duodenum, claudin-2 showed comparable spatial association with LC3B and LAMP1 (Fig. 4r).

In the ileum, claudin-2 staining intensity was unchanged in ethanol-fed mice compared with controls (Fig. 4s–u). Manders' colocalisation analysis showed that claudin-2–LC3B overlap increased significantly (*p* = 0.04) following ethanol exposure (Manders’ coefficient ∼0.05 in controls to ∼0.10 in ethanol-fed mice) (Fig. 4v). Claudin-2–LAMP1 colocalisation was also significantly increased (*p* = 0.04), rising from approximately 0.38 in controls to ∼0.58 after ethanol exposure (Fig. 4w). Within both control and ethanol-fed ileum, claudin-2 association with LAMP1 was significantly greater than its association with LC3B (control, *p* = 0.001; ethanol, *p* < 0.0001; Fig. 4x).

Consistent with the villus analyses, crypt compartments also exhibited spatial association between claudin-2 and autophagy–lysosome markers (Fig. S7). In human ileal crypt epithelium, claudin-2–LC3B overlap remained unchanged between samples from individuals without and with AUD (∼0.5), whereas claudin-2–LAMP1 colocalisation increased significantly (*p* < 0.0001), from ∼0.25 in individuals without AUD to ∼0.6 in individuals with AUD. Within crypts from individuals without AUD, claudin-2 association with LC3B was significantly greater (*p* < 0.0005), than with LAMP1, whereas in crypts from individuals with AUD, claudin-2 showed comparable association with both markers (*p* = 0.21) (Fig. S7a–e). In mouse crypt epithelium, claudin-2 colocalisation with LC3B and LAMP1 remained unchanged in both duodenum (∼0.38–0.42) and ileum (∼0.55–0.85) between control and ethanol-fed animals, and LC3B and LAMP1 associations were similar within both control and ethanol-fed groups (Fig. S7f–o). Statistical comparisons between two groups were performed using two-tailed, unpaired student's *t*-tests.

### ER stress–associated autophagy–lysosome remodelling regulates claudin-2 in the ileum

Given the spatial association between claudin-2 and autophagy–lysosome markers in intestinal epithelium, we examined whether claudin-2 abundance relates to autophagy–lysosome and cellular stress status. Disruption of autophagy–lysosome homoeostasis can induce ER stress and activate STAT3, a regulator of claudin-2 expression, and ER stress–associated proteasome activity may further regulate claudin-2 turnover.^29^ We therefore assessed whether stress signalling and alternative protein degradation pathways converge to regulate stress-responsive claudin-2 expression.

In the duodenum, ethanol feeding decreased (*p* = 0.02) claudin-2 protein levels in the soluble fraction, whereas levels in the insoluble fraction were unchanged (*p* = 0.29) (Fig. 5a and b). Phosphorylated and total STAT3 protein levels were unchanged (*p* = 0.55) (Fig. 5a). Transcript analysis showed no changes in *claudin-2* (*p* = 0.36), *Tnfα* (*p* = 0.20), *Il1β* (*p* = 0.49), or *Il23a* (*p* = 0.23) mRNA expression, whereas *Il6* was significantly increased (*p* = 0.04) (Fig. 5c). ER stress markers, including phosphorylated eIF2α (*p* = 0.40) and CHOP (*p* = 0.43) in soluble fractions, were unchanged following ethanol feeding (Fig. 5g and h), while PDI levels in total homogenates also remained unchanged (*p* = 0.75) (Fig. 5i and j). Trypsin-like (*p* = 0.40) and chymotrypsin-like (*p* = 0.86) proteasome activities were similarly unaltered in the soluble fractions (Fig. 5k).

**Fig. 5:**
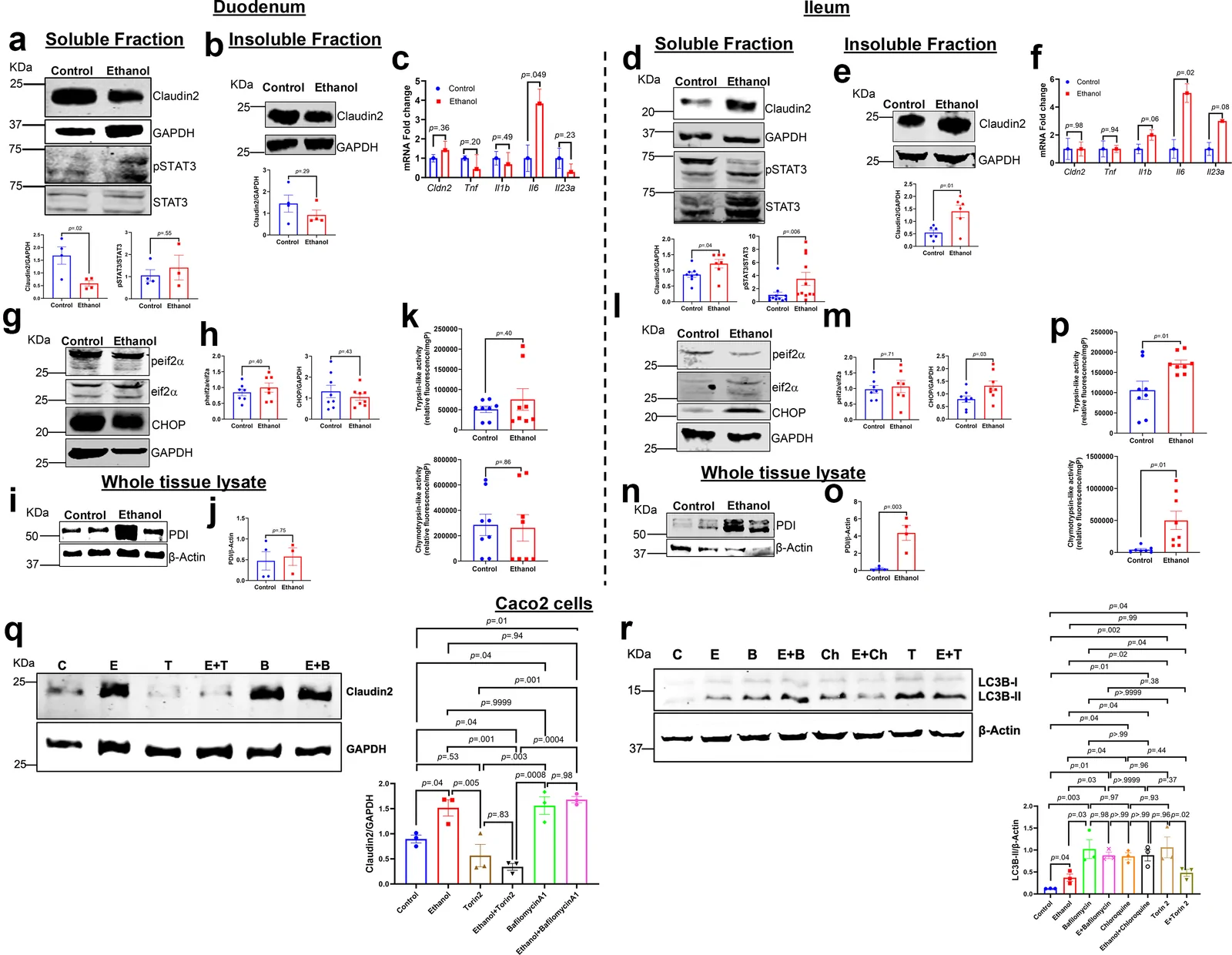
**Ethanol exposure engages ER stress–linked autophagy–lysosome remodelling associated with claudin-2 protein abundance.** Representative immunoblots and densitometric quantification of claudin-2, phosphorylated STAT3 (pSTAT3), and total STAT3 in soluble fractions, and claudin-2 in insoluble fractions, of (**a, b**) duodenal and (**d, e**) ileal mucosa from control- and ethanol-fed mice. (**c**) mRNA expression levels of inflammatory markers in the duodenum and (**f**) ileum from control- and ethanol-fed mice. Representative immunoblots and densitometric quantification of ER stress markers and proteasome activity (trypsin-like and chymotrypsin-like activities) in (**g–k**) duodenum and (**l–p**) ileum from control- and ethanol-fed mice (*n* = 3–4 animals per group). Representative immunoblots and densitometric quantification of (**q**) claudin-2 and (**r**) LC3B-II expression in Caco-2 cells exposed to 50 mM ethanol for 24 h in the presence or absence of the indicated agents: bafilomycin A1 (100 nM, final 4 h), chloroquine (20 μM, 24 h), or Torin-2 (10 nM, 24 h). *n* = 3 independent experiments. Statistical comparisons between two groups were performed using two-tailed, unpaired student's *t*-tests; comparisons involving multiple groups were performed using two-way ANOVA followed by Tukey's post-hoc test.

In contrast, in the ileum, ethanol feeding increased claudin-2 protein levels in both soluble (*p* = 0.04), and insoluble fractions (*p* = 0.01) (Fig. 5d and e) despite unchanged *claudin-*2 mRNA expression (*p* = 0.98) (Fig. 5f). Phosphorylation of STAT3 protein levels increased in the soluble fraction (*p* = 0.006) (Fig. 5d). Transcript analysis showed increased *Il6* expression (*p* = 0.02), unchanged *Tnfα* (*p* = 0.94), and nonsignificant increases in *Il1β* (*p* = 0.06) and *Il23a* (*p* = 0.08). ER stress–associated proteins showed increased CHOP levels (*p* = 0.03) in soluble fractions, while phosphorylated eIF2α remained unchanged (*p* = 0.71) (Fig. 5l and m). In contrast, PDI levels in total homogenates were increased (*p* = 0.003) (Fig. 5n and o). Trypsin-like (*p* = 0.01), and chymotrypsin-like (*p* = 0.01), proteasome activities were also increased in the soluble fractions (Fig. 5p).

To test autophagy–lysosome regulation of claudin-2, we examined claudin-2 protein levels in Caco-2 cells following pharmacologic modulation of autophagy and lysosomal function. Ethanol increased claudin-2 protein levels (*p* = 0.04 vs control), whereas Torin-2, an mTOR inhibitor that induces autophagy, prevented ethanol-induced accumulation (*p* = 0.001 vs ethanol). Inhibition of lysosomal degradation with bafilomycin A1 increased claudin-2 (*p* = 0.04 vs control) with levels not significantly different from those observed with ethanol (*p* = 0.999). Co-treatment with ethanol and bafilomycin A1 did not further increase claudin-2 abundance compared with either ethanol (*p* = 0.94) or bafilomycin A1 alone (*p* = 0.98) (Fig. 5q).

Autophagy flux analysis showed that ethanol increased LC3B-II levels (*p* = 0.04 vs control). Bafilomycin A1 and chloroquine further elevated LC3B-II compared with ethanol alone (*p* = 0.03; *p* = 0.04, respectively), whereas co-treatment did not produce additional LC3B-II accumulation (*p* = 0.98; *p* = 0.99, respectively). Torin-2 increased LC3B-II to levels not significantly different from those observed with the lysosomal inhibitors (*p* > 0.99; *p* = 0.93) whereas ethanol co-treatment reduced Torin-2-induced LC3B-II accumulation to levels not significantly different from ethanol alone (*p* = 0.02; *p* = 0.99) (Fig. 5r). Statistical comparisons between two groups were performed using two-tailed, unpaired student's *t*-tests; comparisons involving multiple groups were performed using two-way ANOVA followed by Tukey's post-hoc test.

### ATG16L1 insufficiency exacerbates ethanol-induced intestinal barrier dysfunction and liver injury

To determine whether impaired autophagosome formation functionally contributes to barrier dysfunction and downstream injury, we next examined whether partial genetic disruption of autophagosome formation alters intestinal barrier function and downstream liver injury using *Atg16l1* hypomorphic mice (*Atg16l1* hm/hm).

Ex vivo measurement of FITC-dextran leakage in freshly isolated ileal segments revealed that ethanol feeding significantly increased ileal permeability in wild-type mice (*p* = 0.0007) (Fig. 6a). Notably, *Atg16l1* hm/hm mice fed a control diet exhibited ileal permeability comparable to that of ethanol-fed wild-type mice. Ethanol feeding further increased ileal permeability in *Atg16l1* hm/hm mice, resulting in an approximately 1.3-fold (*p* = 0.003) increase compared with ethanol-fed wild-type mice.

**Fig. 6:**
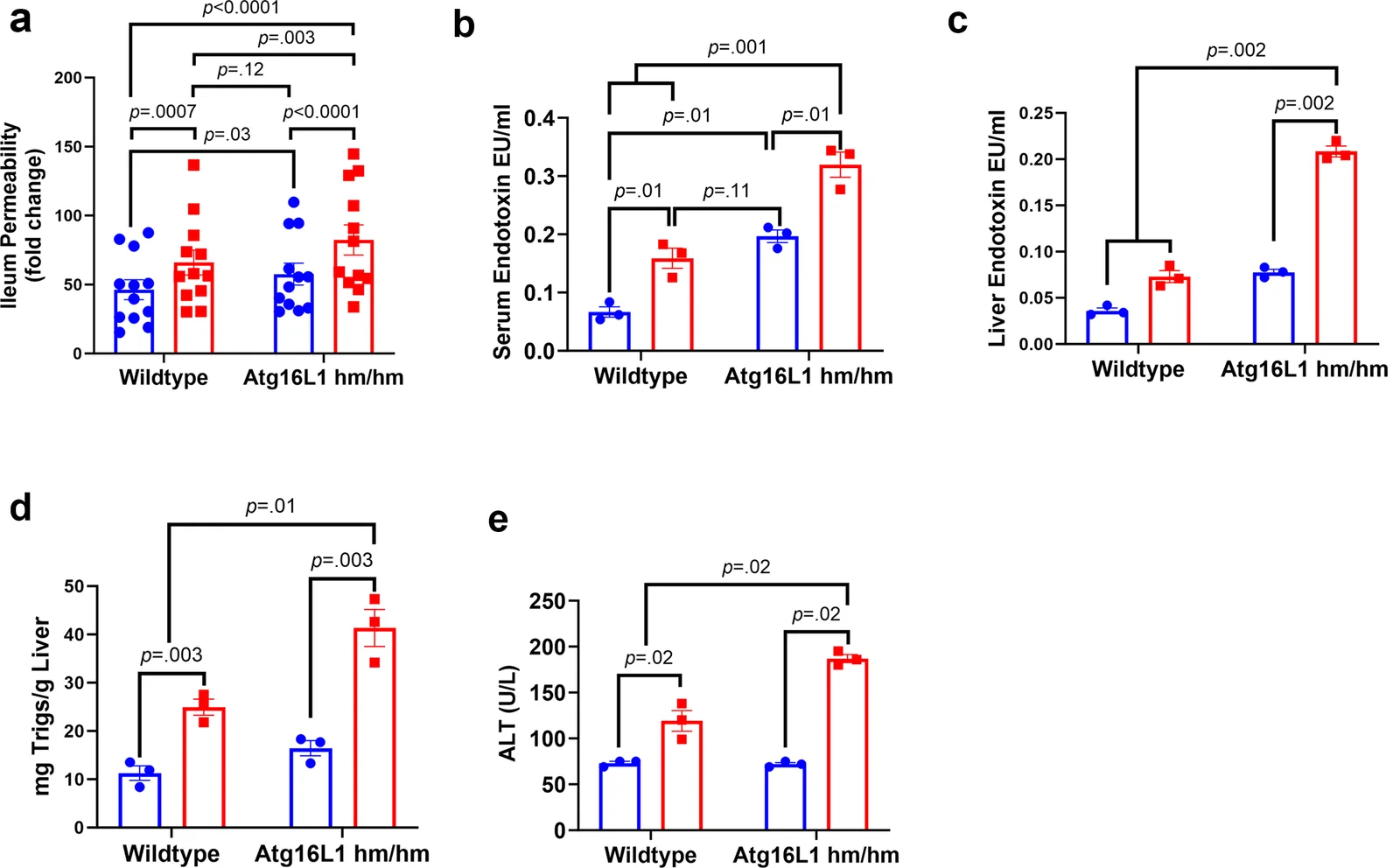
***Atg16l1* hypomorphism is associated with increased intestinal permeability, endotoxemia, and liver injury during ethanol exposure.** (**a**) Ex vivo FITC-dextran permeability in ileal segments; (**b**) serum endotoxin levels; (**c**) hepatic endotoxin levels; (**d**) hepatic triglyceride levels; and (**e**) serum ALT levels in littermate wild-type and ATG16L1ˆhm/hm mice fed control or ethanol diets. Data are presented as mean ± SEM (*n* = 3 animals per group). Statistical significance was determined by two-way ANOVA followed by Tukey's post hoc test.

Consistent with enhanced barrier dysfunction, serum endotoxin levels were increased in ethanol-fed wild-type mice (*p* = 0.01) (Fig. 6b). Control diet-fed *Atg16l1* hm/hm mice displayed serum endotoxin levels comparable to those of ethanol-fed wild-type mice, whereas ethanol feeding further elevated serum endotoxin in *Atg16l1* hm/hm mice, resulting in an approximately 2-fold (*p* = 0.001) increase relative to ethanol-fed wild-type mice. In contrast, hepatic endotoxin levels were comparable between control and ethanol-fed wild-type mice (Fig. 6c). However, control diet-fed *Atg16l1* hm/hm mice exhibited hepatic endotoxin levels comparable to those of wild-type controls, and ethanol-fed, wild-type mice whereas ethanol feeding further increased hepatic endotoxin in *Atg16l1* hm/hm mice, resulting in an approximately 3-fold (*p* = 0.002) increase relative to both control *Atg16l1* hm/hm and ethanol-fed wild-type mice (*p* = 0.002).

Basal hepatic triglyceride and serum ALT levels were comparable between control diet-fed wild-type and *Atg16l1* hm/hm mice (Fig. 6d and e). Ethanol feeding increased hepatic triglycerides and ALT levels in wild-type mice by 2.2-fold (*p* = 0.003) and 1.63-fold (*p* = 0.02), respectively. Importantly, ethanol-fed *Atg16l1* hm/hm mice exhibited greater increases in hepatic triglycerides and ALT, with levels rising by 1.67-fold (*p* = 0.01) and 1.57-fold (*p* = 0.02), respectively, beyond those observed in ethanol-fed wild-type mice. Statistical comparisons were performed using two-way ANOVA followed by Tukey's post-hoc test.

## Discussion

Alcohol induces regionally structured remodelling of the autophagy–lysosome system throughout the intestinal epithelium. Integrating human transcriptomics, tissue imaging, donor-derived organoids, and compartment-resolved mouse analyses, we show that alcohol alters autophagosome formation, lysosomal organisation, and cargo handling in region- (duodenum vs ileum) and compartment-specific (crypt vs villus) patterns. These alterations link transcriptional reprogramming to subcellular and morphological changes. Importantly, this remodelling converges on tight junction regulation and barrier function, identifying autophagy–lysosome pathways as key regulators of intestinal permeability during alcohol exposure.

Previous studies show that ethanol disrupts tight-junction proteins and increases intestinal permeability, yet intracellular mechanisms governing junctional protein handling remain poorly defined.^26^, ^30^ Our findings link defective intracellular trafficking to barrier dysfunction, a central driver of endotoxemia and liver injury in alcohol-associated disease.

Our analysis of human duodenal RNA-seq data from individuals with AUD supports this model. Rather than coordinated induction of canonical autophagy machinery, AUD was associated with preferential expansion of lysosome- and trafficking-related transcriptional programs. In contrast, only a subset of autophagosome-related transcripts were altered, with selective reductions in initiation and membrane-delivery components. This imbalance is consistent with lysosomal stress-adaptive response without proportional engagement of upstream autophagosome biogenesis. Consistent with this interpretation, human ileal tissue from individuals with AUD exhibited increased LC3B and LAMP1 staining, supporting lysosomal remodelling *in situ*, whereas TFEB nuclear, cytoplasmic, and total staining intensities were unchanged. Notably, p62 abundance was reduced in villus epithelium but unchanged in crypts, arguing against uniform impairment in degradation and instead supporting region-specific changes in autophagy flux or cargo handling. Such spatially restricted alterations in epithelial proteostasis may differentially affect barrier integrity along the gut, with implications for regional susceptibility to alcohol-induced permeability and systemic inflammation.

Human intestinal organoid models enabled isolation of epithelial-intrinsic responses to ethanol. At baseline, organoids derived from individuals without and with AUD showed no differences in autophagosome- or lysosome-related transcript abundance, suggesting that the lysosomal adaptive remodelling observed in human intestinal tissue may reflect an *in vivo* adaptation not maintained *ex vivo*, although the use of different donors for tissue and organoid studies limits direct comparison of these findings. However, acute ethanol exposure of organoids from individuals without AUD recapitulated key features seen in human tissue, including selective induction of lysosomal protease transcripts and increased punctate accumulation of LC3B, p62, and LAMP1. These findings demonstrate that ethanol directly engages epithelial autophagy–lysosome pathways independent of systemic influences.

Despite this transcriptional and protein-level responsiveness, a tandem RFP–GFP–LC3B reporter revealed functional differences in autophagy pathway engagement not evident at the mRNA level. Organoids derived from tissue of individuals with AUD exhibited reduced autophagic responsiveness at baseline and an altered distribution of LC3-positive structures upon ethanol exposure, whereas ethanol exposure in organoids derived from individuals without AUD impaired lysosomal maturation without increasing autophagosome formation. Together, these data suggest that ethanol induces lysosomal adaptive remodelling in naïve epithelium, whereas organoids from donors with AUD exhibit a constrained autophagic state that may limit effective throughput during stress. This incomplete adaptation may render the epithelium vulnerable to repeated injury and barrier dysfunction.

Our *in vivo* analyses demonstrate that ethanol induces compartmentalised remodelling of the autophagy–lysosome system along the crypt–villus axis of the mouse duodenum, a dimension obscured in bulk tissue analyses. In differentiated villus epithelial cells, ethanol feeding increased LC3B, p62, and LAMP1 abundance within the epithelium, with LC3B and LAMP1 enriched apically near tight junctions and p62 preferentially localised basally. Ethanol increased the size of p62-and LAMP1-positive puncta without altering LC3B puncta size, indicating selective remodelling of lysosome-associated and cargo-handling compartments. Apical enlargement of LAMP1-positive structures with basal expansion of p62 puncta suggests increased lysosomal processing demand in villus epithelium. These changes are consistent with increased epithelial stress and demand for protein turnover at the luminal interface, where barrier integrity is critical.

Compartment-resolved analyses using tandem RFP–EGFP–LC3B reporter mice further supported engagement of autophagy–lysosome pathways in villus epithelium. Ethanol feeding increased both non-acidic and acidic LC3-positive compartments in villi, consistent with enhanced delivery of LC3-positive structures toward lysosomes. However, levels of free GFP, generated during lysosomal degradation of GFP–LC3, did not increase significantly. Together, lysosomal enlargement and p62 accumulation with unchanged free GFP levels are consistent with increased lysosomal engagement without increased autophagic clearance. This pattern of increased lysosomal engagement without increased autophagic clearance suggests that epithelial cells experience sustained proteostatic stress, which may compromise their ability to maintain tight junction integrity under alcohol exposure.

Immunoblot analyses further clarify the molecular basis of this constrained remodelling. Ethanol feeding increased soluble p62 and LAMP1 abundance while reducing p62 in insoluble fractions, indicating redistribution of p62 from membrane-associated compartments into soluble pools, consistent with altered cargo partitioning between autophagy compartments and cytosolic pools.^31^ In parallel, the ATG12–ATG5 conjugate, required for autophagosome elongation and maturation, was reduced, whereas LC3B-II, ATG7, total ATG16L1, and BECLIN-1 levels remained unchanged. Together, these findings indicate that ethanol-induced lysosomal remodelling in villus epithelium reflects increased cargo handling and lysosomal engagement with constrained autophagosome maturation.

In contrast to villus epithelial cells, crypt epithelium exhibited a distinct response to ethanol exposure. Although LC3B, p62, and LAMP1 staining intensities were increased, ethanol feeding increased the size of p62-and LAMP1-positive puncta without altering LC3B puncta size. Reporter analyses revealed reduced acidic LC3-positive compartments in crypt cells, while non-acidic populations were preserved, indicating impaired autophagosome maturation or delivery to lysosomes rather than enhanced autophagic engagement. Consistent with this pattern, ethanol increased mTOR activation in crypt-enriched fractions, a signalling state known to suppress autophagosome formation and maturation.^32^ These findings suggest that ethanol restrains autophagy–lysosome engagement in proliferative crypt cells. Given the role of crypt cells in epithelial renewal, impaired autophagy in this compartment may limit regenerative capacity and contribute to sustained barrier defects during chronic alcohol exposure.

Transcriptional analyses across crypt-enriched samples and whole duodenal mucosa further reinforce compartment-specific differences in the coordination of lysosomal remodelling and autophagosome biogenesis. In crypt-enriched samples, ethanol altered a limited subset of autophagy- and lysosome-related transcripts without broad induction of core autophagosome biogenesis genes, while whole-tissue analyses similarly revealed modulation of lysosome-associated transcripts over coordinated activation of autophagosome machinery. Collectively, these data indicate compartment-specific lysosomal remodelling without proportional activation of autophagosome biogenesis in the duodenum.

In contrast to the duodenum, the ileum displayed a regulatory state characterised by suppression of autophagosome assembly capacity with preserved or modest lysosomal remodelling. Although LC3B, p62, and LAMP1 accumulated within both villus and crypt compartments following ethanol exposure, puncta size remained unchanged, indicating no overt structural expansion of lysosomal compartments. Instead, biochemical analyses revealed coordinated reductions in key autophagosome assembly proteins, including ATG7, total ATG16L1, and BECLIN-1, together with preserved or modestly increased lysosome-associated components and mTOR activation, particularly in crypt-enriched fractions. These changes occurred primarily in soluble protein pools, while membrane-associated fractions remained unchanged, indicating reduced availability of autophagosome assembly machinery rather than redistribution of existing autophagosome–lysosome compartments.

Compartment-specific autophagic flux analyses using reporter mice further reinforced this restrictive regulatory state. In crypt epithelial cells, ethanol exposure reduced acidified LC3-positive structures while increasing non-acidic LC3-positive intermediates, consistent with impaired progression of LC3-positive structures toward lysosomal degradation with limited autophagosome support. In villus epithelial cells, ethanol exposure increased non-acidified LC3-positive structures without a corresponding increase in acidified compartments. Consistent with this pattern, free GFP levels in ileal mucosa from GFP–LC3B mice trended downward but did not reach statistical significance, providing no evidence for effective autophagic degradative throughput.

Transcriptional analyses further supported this pattern, revealing compartment-specific changes in autophagy- and lysosome-related transcripts without coordinated induction of core autophagosome machinery in crypt-enriched or whole ileal tissue. Together, these findings identify the ileum as a region in which ethanol exposure enforces a constrained autophagy regulatory state, marked by limited autophagosome assembly capacity and restrained lysosomal adaptation that may limit efficient cargo handling in a tissue with high microbial and metabolic demand. This regional vulnerability may be particularly relevant given the high microbial exposure of the ileum, where impaired autophagy–lysosome function may contribute to microbial translocation, endotoxemia, and downstream liver injury. Together with fractionation data, these results suggest that ethanol regulates intestinal autophagy through distinct mechanisms along the gut axis: redistribution of cargo-handling compartments in the duodenum vs restriction of autophagosome assembly capacity in the ileum. The mechanisms underlying these regional and compartment-specific responses remain unclear. Differences in epithelial composition and differentiation state, luminal and microbial exposure, and local inflammatory or stress signalling may contribute to the observed spatial heterogeneity. These factors were not independently resolved in this study, and cell type- and region-resolved studies will be needed to define their contributions.

This dysregulated autophagy–lysosome landscape is associated with altered handling of tight junction proteins, exemplified by claudin-2. Although autophagy-dependent claudin-2 degradation has been described in non-alcohol contexts,^33^, ^34^ our findings support an association between intestinal autophagy–lysosome organisation and claudin-2 localisation and abundance during alcohol exposure. Across models, claudin-2 associates with LC3B- and LAMP1-positive compartments, supporting autophagy–lysosome–dependent regulation. Notably, claudin-2 preferentially localises to lysosome-associated compartments in key epithelial contexts, suggesting a prominent role for lysosomal pathways in its turnover. Given the role of claudin-2 as a pore-forming tight junction protein, altered trafficking and turnover may influence epithelial permeability and may contribute to the “leaky gut” phenotype observed in alcohol-associated disease.

In human ileal epithelium, AUD history was associated with reduced total claudin-2 abundance without detectable changes in its overlap with LC3B or LAMP1, suggesting that lower claudin-2 abundance was not accompanied by a detectable shift in its association with these compartments. In contrast, acute ethanol exposure in human organoids increased claudin-2 association with LAMP1-positive compartments without altering LC3B association or total abundance, consistent with enhanced lysosome-proximal routing rather than depletion of junctional pools*. In vivo*, ethanol feeding produced distinct regional effects: in the ileum, claudin-2 association with both LC3B- and LAMP1-positive compartments increased despite unchanged total abundance, indicating increased association with autophagy–lysosome–associated compartments, whereas in the duodenum ethanol increased claudin-2 abundance without detectable changes in LC3B or LAMP1 association, suggesting junctional induction without altered intracellular trafficking.

Crypt compartments showed largely stable claudin-2 association with LC3B and LAMP1, indicating limited ethanol-induced redistribution of claudin-2 in proliferative crypt epithelium where autophagy activity is constrained. This stability is consistent with the constrained autophagy regulatory state observed in proliferative crypt epithelium. Consistent with this interpretation, claudin-2 mRNA levels remained unchanged, supporting post-transcriptional redistribution into autophagosome-lysosome compartments. Given claudin-2's role as a pore-forming tight junction protein during epithelial stress,^12^, ^33^, ^34^ these data support a model in which autophagy–lysosome pathways may influence claudin-2 turnover and spatial routing within the intestinal epithelium. Together, these findings indicate that alcohol reshapes claudin-2 trafficking according to the regional autophagy regulatory state of the intestinal epithelium.

Despite ethanol-induced remodelling of autophagy–lysosome pathways in the duodenum, lysosomal adaptation appears sufficient to limit secondary activation of ER stress, STAT3 signalling, and proteasome-dependent protein degradation, in contrast to the ileum where reduced autophagosome assembly capacity necessitates engagement of these stress pathways. Consistent with links between autophagy–lysosome disruption and ER stress–dependent STAT3 activation in epithelial systems,^35^ the ileum exhibited induction of ER stress markers, STAT3 activation, and increased proteasome activity alongside constrained autophagosome biogenesis and limited lysosomal adaptation. Enhanced proteasome activity likely reflects increased reliance on cytosolic degradation when autophagy–lysosome capacity is constrained. However, these compensatory pathways are insufficient to maintain epithelial barrier integrity, highlighting the non-redundant role of autophagy–lysosome pathways in gut homoeostasis.

Importantly, this shift toward proteasome-dependent degradation has direct implications for tight junction regulation. In Caco-2 cells, ethanol increased claudin-2 protein levels, an effect recapitulated by lysosomal inhibition (bafilomycin A1). A similar increase in claudin-2 was observed with chloroquine (data not shown). The similar response to two mechanistically distinct inhibitors, both of which disrupt lysosomal cargo degradation, provides convergent evidence that lysosomal inhibition promotes claudin-2 accumulation. These findings support impaired lysosomal processing as a key mechanism underlying claudin-2 accumulation during ethanol exposure.

Conversely, induction of autophagy with Torin-2 reduced claudin-2 levels and prevented ethanol-induced accumulation, supporting the conclusion that claudin-2 delivery to autophagy–lysosome pathways remains intact. However, ethanol attenuated Torin-2–induced LC3B-II accumulation, indicating that ethanol limits effective autophagosome maturation and turnover. Together, these findings support a model in which ethanol permits claudin-2 entry into autophagy-associated compartments but limits lysosome-dependent processing, leading to reduced turnover of junctional proteins required for adaptive barrier remodelling.

While proteasome inhibition stabilised claudin-2 levels in Caco-2 cells (data not shown), ER-associated and proteasome-based quality-control pathways are poorly suited for the rapid, spatially restricted turnover of junctional proteins, which relies on endocytic trafficking and lysosome-associated processing.^36^ *In vivo*, the apical positioning of lysosome-associated compartments in villus epithelial cells places the autophagy–lysosome system in close proximity to tight junction complexes, supporting localised regulation of junctional protein availability during epithelial stress. Together, these findings indicate that when autophagy–lysosome coordination is disrupted in the ileum, compensatory ER stress and proteasome responses are insufficient to replace lysosome-dependent handling of tight junction proteins, potentially contributing to dysregulated claudin-2 turnover and impaired barrier-adaptive remodelling during ethanol exposure. These findings provide a potential explanation for how regional differences in autophagy–lysosome regulation may alter tight junction protein turnover and contribute to compromised intestinal homoeostasis in alcohol-associated disease.

The functional importance of intact autophagosome biogenesis within this framework is underscored by our studies in *Atg16l1* hypomorphic mice, which provide genetic evidence for the consequences of limiting autophagosome formation. Partial disruption of autophagosome biogenesis was sufficient to increase baseline intestinal permeability and markedly exacerbate ethanol-induced barrier dysfunction, endotoxemia, and liver injury. These findings demonstrate that impaired autophagosome biogenesis can exacerbate gut barrier dysfunction and gut–liver axis pathology during alcohol exposure. Importantly, they demonstrate that compensatory proteostasis pathways are insufficient to maintain epithelial homoeostasis in the absence of coordinated autophagosome–lysosome flux, thereby providing *in vivo* functional support for the disrupted autophagy–lysosome coordination observed in our human, organoid, and compartment-resolved intestinal analyses. However, because *Atg16l1* hypomorphism is global, the contribution of altered immune-cell autophagy to the systemic inflammatory phenotype cannot be excluded. The relative contributions of epithelial and immune-cell autophagy dysfunction to alcohol-induced barrier dysfunction and systemic inflammation therefore require further investigation.

While these findings provide complementary evidence across human, organoid, and animal models, several limitations should be considered. The human cohorts were relatively small and not population-based, and the tissue and organoid studies involved independent, unmatched donors with limited demographic and clinical information. These factors limit comparability and generalisability and may introduce residual confounding that could not be reliably addressed by statistical matching or multivariable adjustment. Although mice were weight-matched, allocation was not formally randomised, so pre-existing differences between groups cannot be excluded as contributing factors to the observed outcomes. In addition, individual animal experiments used a single sex, with female mice used for most studies and male mice used for the *Atg16l1* hypomorphic studies. The *Atg16l1* phenotype was assessed against sex-matched male wild-type controls and was not directly compared with the female experimental cohorts; however, whether this phenotype differs by sex remains unknown. Small sample sizes in the mechanistic mouse experiments may have limited detection of modest effects.

In summary, our findings identify spatially heterogeneous disruption of autophagy–lysosome coordination along the intestinal epithelium. Alcohol exposure is associated with lysosomal stress/adaptive remodelling occurring alongside limited autophagosome formation, maturation, and autophagic throughput, with responses varying by epithelial compartment (crypt vs villus) and intestinal region. Preserved cargo targeting occurs alongside constrained autophagic throughput, leading to altered intracellular trafficking and limited degradative processing. This dysregulated autophagy–lysosome landscape is associated with altered tight junction protein handling, while impaired autophagosome biogenesis exacerbates epithelial barrier dysfunction, endotoxemia, and liver injury. Collectively, these results position intestinal autophagy–lysosome coordination as a spatially organised epithelial stress-response system whose disruption by alcohol compromises barrier function and contributes to gut–liver disease progression. The human findings support the relevance of these pathways to AUD, while their broader applicability requires validation in larger, well-characterised cohorts.

## Contributors

M.A. was responsible for study design and for the acquisition, analysis, and interpretation of data, as well as drafting and editing the manuscript. A.B. contributed to data acquisition, analysis, and interpretation. J.D.D. assisted with studies involving ATG16L1ˆhm/hm mice and provided scientific guidance and technical support. A.K. contributed to human tissue studies, experimental design, and scientific input. N.V.N.T. and T.N. performed RNA-seq data reanalysis and data visualisation; T.N. also provided scientific input. P.G.T. conceived and designed the study, supervised the research, interpreted the data, and drafted and edited the manuscript. M.A. and P.G.T. accessed and verified the underlying data. All authors read and approved the final version of the manuscript prior to submission.

## Data sharing statement

All data supporting this study are included in the Article and its Supplementary Information. Previously published RNA-seq data analysed here are available from the cited source.

## Declaration of interests

The authors declare that the research was conducted with no commercial or financial relationships that could be construed as potential conflicts of interest.

## Appendix A Supplementary data

Supplementary Tabl es

## References

[1] Simon L., Souza-Smith F.M., Molina P.E. (2022). Alcohol-associated tissue injury: current views on pathophysiological mechanisms. Annu Rev Physiol.

[2] Souza-Smith F.M., Lang C.H., Nagy L.E., Bailey S.M., Parsons L.H., Murray G.J. (2016). Physiological processes underlying organ injury in alcohol abuse. Am J Physiol Endocrinol Metab.

[3] Yokus B., Maccioni L., Fu L. (2026). The link between alcohol consumption and kidney injury. Am J Pathol.

[4] Li Z., Gu M., Zaparte A. (2024). Alcohol-induced gut microbial reorganization and associated overproduction of phenylacetylglutamine promotes cardiovascular disease. Nat Commun.

[5] Osna N.A., Rasineni K., Ganesan M., Donohue T.M., Kharbanda K.K. (2022). Pathogenesis of alcohol-associated liver disease. J Clin Exp Hepatol.

[6] Gao H., Jiang Y., Zeng G. (2024). Cell-to-cell and organ-to-organ crosstalk in the pathogenesis of alcohol-associated liver disease. eGastroenterology.

[7] Fu Y., Mackowiak B., Lin Y.H. (2024). Coordinated action of a gut-liver pathway drives alcohol detoxification and consumption. Nat Metab.

[8] Bishehsari F., Magno E., Swanson G. (2017). Alcohol and gut-derived inflammation. Alcohol Res.

[9] Hammer A.M., Morris N.L., Earley Z.M., Choudhry M.A. (2015). The first line of defense: the effects of alcohol on post-burn intestinal barrier, immune cells, and microbiome. Alcohol Res.

[10] Chancharoenthana W., Kamolratanakul S., Udompornpitak K., Wannigama D.L., Schultz M.J., Leelahavanichkul A. (2025). Alcohol-induced gut permeability defect through dysbiosis and enterocytic mitochondrial interference causing pro-inflammatory macrophages in a dose dependent manner. Sci Rep.

[11] Maccioni L., Leclercq I.A., Schnabl B., Starkel P. (2021). Host factors in dysregulation of the gut barrier function during alcohol-associated liver disease. Int J Mol Sci.

[12] Fernandez A.L., Koval M., Fan X., Guidot D.M. (2007). Chronic alcohol ingestion alters claudin expression in the alveolar epithelium of rats. Alcohol.

[13] Liu S., Yao S., Yang H., Liu S., Wang Y. (2023). Autophagy: regulator of cell death. Cell Death Dis.

[14] Naama M., Telpaz S., Awad A. (2023). Autophagy controls mucus secretion from intestinal goblet cells by alleviating ER stress. Cell Host Microbe.

[15] Cadwell K., Liu J.Y., Brown S.L. (2008). A key role for autophagy and the autophagy gene Atg16l1 in mouse and human intestinal Paneth cells. Nature.

[16] Ganapathy A.S., Saha K., Wang A. (2023). Alpha-tocopherylquinone differentially modulates claudins to enhance intestinal epithelial tight junction barrier via AhR and Nrf2 pathways. Cell Rep.

[17] Nighot P.K., Hu C.A., Ma T.Y. (2015). Autophagy enhances intestinal epithelial tight junction barrier function by targeting claudin-2 protein degradation. J Biol Chem.

[18] Saha K., Subramenium Ganapathy A., Wang A. (2023). Autophagy reduces the degradation and promotes membrane localization of occludin to enhance the intestinal epithelial tight junction barrier against paracellular macromolecule flux. J Crohns Colitis.

[19] Bakheet A., Anbazhagan M., Barve S. (2025). Perspective on alcohol-induced organ damage via autophagy-dependent cellular changes. Redox Biol.

[20] Bohn M.J., Babor T.F., Kranzler H.R. (1995). The alcohol use disorders identification test (AUDIT): validation of a screening instrument for use in medical settings. J Stud Alcohol.

[21] Bertola A., Mathews S., Ki S.H., Wang H., Gao B. (2013). Mouse model of chronic and binge ethanol feeding (the NIAAA model). Nat Protoc.

[22] Charan J., Kantharia N.D. (2013). How to calculate sample size in animal studies?. J Pharmacol Pharmacother.

[23] Niklinska-Schirtz B.J., Venkateswaran S., Anbazhagan M. (2021). Ileal derived organoids from crohn's disease patients show unique transcriptomic and secretomic signatures. Cell Mol Gastroenterol Hepatol.

[24] Thomes P.G., Osna N.A., Bligh S.M., Tuma D.J., Kharbanda K.K. (2015). Role of defective methylation reactions in ethanol-induced dysregulation of intestinal barrier integrity. Biochem Pharmacol.

[25] Rao R.K. (2008). Acetaldehyde-induced barrier disruption and paracellular permeability in Caco-2 cell monolayer. Methods Mol Biol.

[26] Meena A.S., Shukla P.K., Bell B. (2022). TRPV6 channel mediates alcohol-induced gut barrier dysfunction and systemic response. Cell Rep.

[27] Kaizuka T., Morishita H., Hama Y. (2016). An autophagic flux probe that releases an internal control. Mol Cell.

[28] Shen L., Weber C.R., Raleigh D.R., Yu D., Turner J.R. (2011). Tight junction pore and leak pathways: a dynamic duo. Annu Rev Physiol.

[29] Nakashima A., Cheng S.B., Kusabiraki T. (2019). Endoplasmic reticulum stress disrupts lysosomal homeostasis and induces blockade of autophagic flux in human trophoblasts. Sci Rep.

[30] Rao R.K., Basuroy S., Rao V.U., Karnaky K.J., Gupta A. (2002). Tyrosine phosphorylation and dissociation of occludin-ZO-1 and E-cadherin-beta-catenin complexes from the cytoskeleton by oxidative stress. Biochem J.

[31] Hinz K., Qian H., Peiffer B., Sun Z., Ni H.M., Ding W.X. (2025). Role of SQSTM1/p62 in regulating Mallory-Denk body in alcohol-associated liver disease. eGastroenterology.

[32] Foerster E.G., Mukherjee T., Cabral-Fernandes L., Rocha J.D.B., Girardin S.E., Philpott D.J. (2022). How autophagy controls the intestinal epithelial barrier. Autophagy.

[33] Ahmad R., Kumar B., Tamang R.L., Talmon G.A., Dhawan P., Singh A.B. (2023). P62/SQSTM1 binds with claudin-2 to target for selective autophagy in stressed intestinal epithelium. Commun Biol.

[34] Ganapathy A.S., Saha K., Suchanec E. (2022). AP2M1 mediates autophagy-induced CLDN2 (claudin 2) degradation through endocytosis and interaction with LC3 and reduces intestinal epithelial tight junction permeability. Autophagy.

[35] You L., Wang Z., Li H. (2015). The role of STAT3 in autophagy. Autophagy.

[36] Shen L., Weber C.R., Turner J.R. (2008). The tight junction protein complex undergoes rapid and continuous molecular remodeling at steady state. J Cell Biol.

