# Supplementary material for: Alcohol disrupts spatial coordination of autophagy–lysosome pathways to impair intestinal barrier integrity
Source: eBioMedicine. 2026 Oct 26;133:106534. doi: 10.1016/j.ebiom.2026.106534 (PMC13734950; doi:10.1016/j.ebiom.2026.106534)
Supplement: Supplementary file 1 [file mmc1.docx]

Supplementary Table

Table S2: Transcripts related to autophagasome

| **SYMBOL** | **Primary_Category** | **Subcategory** | **Direction** | **log2FC** | **p_value** | **p_adjusted** | **t** | **B** | **AUD_mean** | **Control_mean** |
| --- | --- | --- | --- | --- | --- | --- | --- | --- | --- | --- |
| GABARAP | Autophagosome | Core autophagy machinery / receptors | Upregulated | 0.098977228 | 0.001045481 | 0.02890677 | 3.442294962 | -0.950541572 | 6.942817585 | 6.843840357 |
| DYNLL2 | Autophagosome | Trafficking/sorting (other) | Upregulated | 0.119345733 | 0.001224287 | 0.02890677 | 3.391026139 | -1.093265636 | 6.789278589 | 6.669932857 |
| ATG101 | Autophagosome | Core autophagy machinery / receptors | Upregulated | 0.177469501 | 0.003700578 | 0.053741336 | 3.018417503 | -2.085770886 | 3.911095613 | 3.733626112 |
| ATG4A | Autophagosome | Core autophagy machinery / receptors | Upregulated | 0.187993054 | 0.009341085 | 0.082707523 | 2.684148811 | -2.903540742 | 4.629234381 | 4.441241328 |
| DYNLL1 | Autophagosome | Trafficking/sorting (other) | Upregulated | 0.159971466 | 0.015516056 | 0.098422741 | 2.489708843 | -3.344757235 | 6.087969888 | 5.927998423 |
| PIK3R4 | Autophagosome | Core autophagy machinery / receptors | Upregulated | 0.074187488 | 0.023429229 | 0.12512761 | 2.324545218 | -3.698374481 | 5.460915073 | 5.386727585 |
| SPATA18 | Autophagosome | Core autophagy machinery / receptors | Upregulated | 0.283996401 | 0.030575143 | 0.131804141 | 2.213832528 | -3.924102662 | 2.926453465 | 2.642457064 |
| ATG5 | Autophagosome | Core autophagy machinery / receptors | Upregulated | 0.074967562 | 0.037214833 | 0.145982512 | 2.129815128 | -4.089175462 | 5.290403323 | 5.215435761 |
| ATG4B | Autophagosome | Core autophagy machinery / receptors | Upregulated | 0.176855492 | 0.049095791 | 0.173880925 | 2.007684221 | -4.319324916 | 6.145238022 | 5.96838253 |
| TAX1BP1 | Autophagosome | Core autophagy machinery / receptors | Upregulated | 0.072625408 | 0.049992191 | 0.175592406 | 1.99954719 | -4.33423962 | 6.63430091 | 6.561675502 |
| ATG9B | Autophagosome | Core autophagy machinery / receptors | Downregulated | -0.381272985 | 0.008825201 | 0.081177895 | -2.705372614 | -2.853801665 | 1.330097776 | 1.711370761 |
| ULK2 | Autophagosome | Core autophagy machinery / receptors | Downregulated | -0.145440704 | 0.02081283 | 0.124547144 | -2.372738586 | -3.597253064 | 3.740720926 | 3.88616163 |
| ATG16L2 | Autophagosome | Core autophagy machinery / receptors | Downregulated | -0.154202746 | 0.023106917 | 0.12512761 | -2.330215224 | -3.686566712 | 5.773788954 | 5.9279917 |

Table S3: Transcripts related to Lysosome

| **SYMBOL** | **Primary_Category** | **Subcategory** | **Direction** | **log2FC** | **p_value** | **p_adjusted** | **t** | **B** | **AUD_mean** | **Control_mean** |
| --- | --- | --- | --- | --- | --- | --- | --- | --- | --- | --- |
| ATP6V1D | Lysosome | Acidification (V-ATPase) | Upregulated | 0.243558217 | 0.000429116 | 0.02890677 | 3.72406287 | -0.141619251 | 5.107610267 | 4.86405205 |
| HSPA8 | Lysosome | Proteostasis/chaperone network | Upregulated | 0.20288223 | 0.001021648 | 0.02890677 | 3.449747784 | -0.929675988 | 9.951767041 | 9.74888481 |
| CTSA | Lysosome | Hydrolases / lumen enzymes | Upregulated | 0.191517259 | 0.001815116 | 0.038571216 | 3.261201725 | -1.44818994 | 7.267933441 | 7.076416182 |
| GNPTG | Lysosome | M6P targeting/trafficking to lysosome | Upregulated | 0.121315442 | 0.002215672 | 0.042802751 | 3.194326797 | -1.627286539 | 4.974592421 | 4.853276979 |
| LAMP1 | Lysosome | Membrane/identity proteins | Upregulated | 0.141588521 | 0.002602772 | 0.044686705 | 3.139728418 | -1.771571751 | 7.789100838 | 7.647512318 |
| ATP6V0D1 | Lysosome | Acidification (V-ATPase) | Upregulated | 0.173069944 | 0.002677027 | 0.044686705 | 3.130133577 | -1.796745577 | 6.26387287 | 6.090802926 |
| GBA | Lysosome | Hydrolases / lumen enzymes | Upregulated | 0.129065533 | 0.003512379 | 0.053312901 | 3.036590224 | -2.039273046 | 5.331867114 | 5.20280158 |
| ATP6V1H | Lysosome | Acidification (V-ATPase) | Upregulated | 0.127973457 | 0.005544158 | 0.065451871 | 2.875379769 | -2.444551358 | 4.296121889 | 4.168148432 |
| CLN3 | Lysosome | Membrane/identity proteins | Upregulated | 0.114314734 | 0.007168052 | 0.077289635 | 2.782215422 | -2.67118468 | 4.897711211 | 4.783396477 |
| GLMP | Lysosome | Membrane/identity proteins | Upregulated | 0.189445733 | 0.007274319 | 0.077289635 | 2.776821042 | -2.684132825 | 5.36466595 | 5.175220217 |
| FUCA1 | Lysosome | Hydrolases / lumen enzymes | Upregulated | 0.161561601 | 0.007486264 | 0.07760152 | 2.766275044 | -2.709390726 | 6.742203574 | 6.580641972 |
| ATP6V0E1 | Lysosome | Acidification (V-ATPase) | Upregulated | 0.086463451 | 0.008355493 | 0.081177895 | 2.725708632 | -2.805857728 | 5.396426967 | 5.309963516 |
| NAGPA | Lysosome | M6P targeting/trafficking to lysosome | Upregulated | 0.201015957 | 0.013355408 | 0.091298323 | 2.548122349 | -3.214985829 | 4.072805131 | 3.871789174 |
| SLC9A6 | Lysosome | Transporters/channels | Upregulated | 0.132925048 | 0.013533634 | 0.091298323 | 2.542992098 | -3.226479945 | 5.241161347 | 5.108236299 |
| ATP13A2 | Lysosome | Transporters/channels | Upregulated | 0.11460709 | 0.016971004 | 0.104531546 | 2.454382186 | -3.42205804 | 5.004222549 | 4.889615459 |
| ATP6V1B2 | Lysosome | Acidification (V-ATPase) | Upregulated | 0.106407649 | 0.024295595 | 0.12512761 | 2.309641177 | -3.729297984 | 6.415033658 | 6.308626009 |
| PDCD6 | Lysosome | ESCRT / membrane remodeling | Upregulated | 0.092534767 | 0.024333496 | 0.12512761 | 2.309000008 | -3.730624591 | 6.308385801 | 6.215851034 |
| ARSA | Lysosome | Hydrolases / lumen enzymes | Upregulated | 0.161886639 | 0.024436686 | 0.12512761 | 2.307258835 | -3.734225611 | 5.688731339 | 5.5268447 |
| ATP6V0C | Lysosome | Acidification (V-ATPase) | Upregulated | 0.119360083 | 0.033742729 | 0.139229708 | 2.171943644 | -4.007082972 | 6.898259157 | 6.778899074 |
| GLB1 | Lysosome | Hydrolases / lumen enzymes | Upregulated | 0.092148836 | 0.034838859 | 0.142370338 | 2.158249793 | -4.033917587 | 5.905603372 | 5.813454536 |
| ATP6V1E1 | Lysosome | Acidification (V-ATPase) | Upregulated | 0.080420124 | 0.038587087 | 0.147743352 | 2.114106703 | -4.119432954 | 5.125819706 | 5.045399582 |
| SLC17A5 | Lysosome | Transporters/channels | Upregulated | 0.128841001 | 0.042475075 | 0.158350059 | 2.072100891 | -4.199397635 | 5.445714142 | 5.316873141 |
| HEXB | Lysosome | Hydrolases / lumen enzymes | Upregulated | 0.10741653 | 0.044178707 | 0.161776952 | 2.05473855 | -4.232044941 | 6.368855965 | 6.261439435 |
| CTSC | Lysosome | Hydrolases / lumen enzymes | Downregulated | -0.359087977 | 0.001071563 | 0.02890677 | -3.434321406 | -0.972832013 | 7.181126698 | 7.540214675 |
| CTSB | Lysosome | Hydrolases / lumen enzymes | Downregulated | -0.160897651 | 0.004580033 | 0.061626519 | -2.943494047 | -2.275310182 | 8.048263343 | 8.209160994 |
| ARSB | Lysosome | Hydrolases / lumen enzymes | Downregulated | -0.231702807 | 0.014801263 | 0.096288072 | -2.508172123 | -3.304001067 | 4.011677945 | 4.243380752 |
| CLN6 | Lysosome | Membrane/identity proteins | Downregulated | -0.137588461 | 0.018781115 | 0.114028198 | -2.414051154 | -3.509209002 | 4.700848291 | 4.838436752 |
| SLC15A3 | Lysosome | Transporters/channels | Downregulated | -0.240815384 | 0.022010897 | 0.12512761 | -2.350037621 | -3.645099176 | 3.662129813 | 3.902945198 |
| MAN2B1 | Lysosome | Hydrolases / lumen enzymes | Downregulated | -0.122711777 | 0.027789737 | 0.131804141 | -2.25395198 | -3.843372525 | 6.308233145 | 6.430944922 |
| IFI30 | Lysosome | Hydrolases / lumen enzymes | Downregulated | -0.169736231 | 0.036986348 | 0.145982512 | -2.132479524 | -4.08402428 | 5.196036587 | 5.365772818 |

Table S4: Transcripts related to Autophagasome-Lysosome Interface

| **SYMBOL** | **Primary_Category** | **Subcategory** | **Direction** | **log2FC** | **p_value** | **p_adjusted** | **t** | **B** | **AUD_mean** | **Control_mean** |
| --- | --- | --- | --- | --- | --- | --- | --- | --- | --- | --- |
| CHMP2A | Autophagosome-Lysosome Interface | ESCRT / membrane remodeling | Upregulated | 0.228153216 | 1.41312E-05 | 0.005456559 | 4.720280702 | 2.999468066 | 5.431231247 | 5.203078031 |
| BLOC1S1 | Autophagosome-Lysosome Interface | Adaptor complexes & lysosome biogenesis trafficking | Upregulated | 0.228224189 | 2.56779E-05 | 0.005456559 | 4.553214967 | 2.446688356 | 4.535965428 | 4.307741239 |
| VPS52 | Autophagosome-Lysosome Interface | Trafficking/sorting (other) | Upregulated | 0.168332714 | 0.000355508 | 0.02890677 | 3.782152149 | 0.030067558 | 6.02382888 | 5.855496166 |
| CHMP3 | Autophagosome-Lysosome Interface | ESCRT / membrane remodeling | Upregulated | 0.11284378 | 0.000369371 | 0.02890677 | 3.770381594 | -0.004851427 | 6.669545506 | 6.556701725 |
| YKT6 | Autophagosome-Lysosome Interface | Fusion SNAREs & cofactors | Upregulated | 0.164484194 | 0.000544266 | 0.02890677 | 3.649997967 | -0.358147212 | 6.067157434 | 5.902673239 |
| DCTN1 | Autophagosome-Lysosome Interface | Motors/tethers/scaffolds | Upregulated | 0.107464803 | 0.000704667 | 0.02890677 | 3.568603408 | -0.592948175 | 6.836269064 | 6.728804261 |
| GOSR1 | Autophagosome-Lysosome Interface | Fusion SNAREs & cofactors | Upregulated | 0.101854592 | 0.000750239 | 0.02890677 | 3.548704258 | -0.649838165 | 6.225211617 | 6.123357025 |
| SNX19 | Autophagosome-Lysosome Interface | Endosomal sorting (Retromer/SNX) | Upregulated | 0.114942629 | 0.000798745 | 0.02890677 | 3.528749759 | -0.706680754 | 7.228536626 | 7.113593997 |
| KIF3B | Autophagosome-Lysosome Interface | Motors/tethers/scaffolds | Upregulated | 0.222863174 | 0.000966475 | 0.02890677 | 3.467653798 | -0.879423234 | 6.942964994 | 6.72010182 |
| SORT1 | Autophagosome-Lysosome Interface | Endosomal sorting (Retromer/SNX) | Upregulated | 0.129035483 | 0.001218082 | 0.02890677 | 3.392682771 | -1.088676176 | 7.451959398 | 7.322923915 |
| VPS53 | Autophagosome-Lysosome Interface | Trafficking/sorting (other) | Upregulated | 0.11770771 | 0.001717229 | 0.038411705 | 3.279654038 | -1.398320974 | 6.962535054 | 6.844827343 |
| RAB7A | Autophagosome-Lysosome Interface | Rab/Arl trafficking & effectors | Upregulated | 0.109261176 | 0.00191166 | 0.038688355 | 3.243898382 | -1.494777011 | 7.212845644 | 7.103584469 |
| RAB1B | Autophagosome-Lysosome Interface | Rab/Arl trafficking & effectors | Upregulated | 0.138419041 | 0.002679643 | 0.044686705 | 3.12980008 | -1.79761958 | 7.231076124 | 7.092657083 |
| PDCD6IP | Autophagosome-Lysosome Interface | ESCRT / membrane remodeling | Upregulated | 0.13766625 | 0.002906272 | 0.04574687 | 3.1020088 | -1.870219315 | 7.970012153 | 7.832345903 |
| VPS33A | Autophagosome-Lysosome Interface | Tethering (HOPS/CORVET) | Upregulated | 0.096986754 | 0.004696471 | 0.061626519 | 2.934597297 | -2.297583148 | 4.768636891 | 4.671650137 |
| AP3S1 | Autophagosome-Lysosome Interface | Adaptor complexes & lysosome biogenesis trafficking | Upregulated | 0.15152679 | 0.004785118 | 0.061626519 | 2.927960065 | -2.314166808 | 5.767905132 | 5.616378342 |
| KIF5B | Autophagosome-Lysosome Interface | Motors/tethers/scaffolds | Upregulated | 0.110343349 | 0.008256295 | 0.081177895 | 2.730137041 | -2.795380483 | 7.903554903 | 7.793211554 |
| CHMP5 | Autophagosome-Lysosome Interface | ESCRT / membrane remodeling | Upregulated | 0.1351186 | 0.008860788 | 0.081177895 | 2.703872597 | -2.857327044 | 5.563156267 | 5.428037667 |
| AP1G1 | Autophagosome-Lysosome Interface | Adaptor complexes & lysosome biogenesis trafficking | Upregulated | 0.086820897 | 0.00897732 | 0.081177895 | 2.698998835 | -2.868770987 | 7.518780441 | 7.431959543 |
| VPS4B | Autophagosome-Lysosome Interface | ESCRT / membrane remodeling | Upregulated | 0.094090018 | 0.009796398 | 0.08489716 | 2.666289082 | -2.94515893 | 6.435035578 | 6.34094556 |
| SNX3 | Autophagosome-Lysosome Interface | Endosomal sorting (Retromer/SNX) | Upregulated | 0.093461429 | 0.010014329 | 0.08489716 | 2.658007424 | -2.964383698 | 6.381214125 | 6.287752696 |
| ARL8A | Autophagosome-Lysosome Interface | Rab/Arl trafficking & effectors | Upregulated | 0.134347289 | 0.010187659 | 0.08489716 | 2.65153729 | -2.979370613 | 4.605010685 | 4.470663396 |
| VPS26B | Autophagosome-Lysosome Interface | Endosomal sorting (Retromer/SNX) | Upregulated | 0.084850255 | 0.011664414 | 0.090976457 | 2.600151151 | -3.097374743 | 5.911288109 | 5.826437854 |
| VPS18 | Autophagosome-Lysosome Interface | Tethering (HOPS/CORVET) | Upregulated | 0.094217951 | 0.011851775 | 0.090976457 | 2.594060738 | -3.111239785 | 4.556576109 | 4.462358159 |
| AP3D1 | Autophagosome-Lysosome Interface | Adaptor complexes & lysosome biogenesis trafficking | Upregulated | 0.114954615 | 0.011987486 | 0.090976457 | 2.589703652 | -3.121143013 | 7.263198444 | 7.148243829 |
| AP1B1 | Autophagosome-Lysosome Interface | Adaptor complexes & lysosome biogenesis trafficking | Upregulated | 0.12821693 | 0.012356854 | 0.091298323 | 2.578067858 | -3.147525181 | 7.148447182 | 7.020230252 |
| HPS5 | Autophagosome-Lysosome Interface | Adaptor complexes & lysosome biogenesis trafficking | Upregulated | 0.253782862 | 0.013065183 | 0.091298323 | 2.556610709 | -3.195927328 | 5.707460136 | 5.453677273 |
| HOOK1 | Autophagosome-Lysosome Interface | Motors/tethers/scaffolds | Upregulated | 0.152183087 | 0.013475281 | 0.091298323 | 2.544665008 | -3.222733897 | 6.257706119 | 6.105523032 |
| VAMP8 | Autophagosome-Lysosome Interface | Fusion SNAREs & cofactors | Upregulated | 0.165549581 | 0.014952971 | 0.096288072 | 2.504187212 | -3.312817985 | 5.821699634 | 5.656150053 |
| VPS4A | Autophagosome-Lysosome Interface | ESCRT / membrane remodeling | Upregulated | 0.066634932 | 0.023875802 | 0.12512761 | 2.316802811 | -3.714459395 | 6.348172221 | 6.281537289 |
| VAMP4 | Autophagosome-Lysosome Interface | Fusion SNAREs & cofactors | Upregulated | 0.090028399 | 0.02409147 | 0.12512761 | 2.313109686 | -3.722116163 | 4.624026234 | 4.533997834 |
| AP3B1 | Autophagosome-Lysosome Interface | Adaptor complexes & lysosome biogenesis trafficking | Upregulated | 0.064497842 | 0.02491974 | 0.126082018 | 2.29919385 | -3.75087572 | 6.42917456 | 6.364676718 |
| AP1M2 | Autophagosome-Lysosome Interface | Adaptor complexes & lysosome biogenesis trafficking | Upregulated | 0.146069877 | 0.026209889 | 0.131049444 | 2.278314425 | -3.793754861 | 5.469395347 | 5.32332547 |
| WASHC2C | Autophagosome-Lysosome Interface | Adaptor complexes & lysosome biogenesis trafficking | Upregulated | 0.114637041 | 0.027462959 | 0.131804141 | 2.25888883 | -3.833354079 | 5.721871769 | 5.607234728 |
| TRIM32 | Autophagosome-Lysosome Interface | Immune/innate regulators linked to autophagy | Upregulated | 0.142313295 | 0.028003066 | 0.131804141 | 2.250756682 | -3.849846985 | 4.500224336 | 4.357911041 |
| CHMP1A | Autophagosome-Lysosome Interface | ESCRT / membrane remodeling | Upregulated | 0.16258018 | 0.029467578 | 0.131804141 | 2.229383297 | -3.89295583 | 6.412092308 | 6.249512128 |
| VAMP7 | Autophagosome-Lysosome Interface | Fusion SNAREs & cofactors | Upregulated | 0.089607274 | 0.029876435 | 0.131804141 | 2.223583723 | -3.904593398 | 5.498768138 | 5.409160864 |
| AP1S1 | Autophagosome-Lysosome Interface | Adaptor complexes & lysosome biogenesis trafficking | Upregulated | 0.242511009 | 0.030006862 | 0.131804141 | 2.221748343 | -3.908270986 | 5.176869367 | 4.934358359 |
| VTI1B | Autophagosome-Lysosome Interface | Fusion SNAREs & cofactors | Upregulated | 0.075211182 | 0.030299924 | 0.131804141 | 2.217649835 | -3.916473987 | 6.274559491 | 6.199348309 |
| PAFAH1B1 | Autophagosome-Lysosome Interface | Motors/tethers/scaffolds | Upregulated | 0.115186452 | 0.030421326 | 0.131804141 | 2.215962232 | -3.919847937 | 7.908770033 | 7.793583582 |
| HPS3 | Autophagosome-Lysosome Interface | Adaptor complexes & lysosome biogenesis trafficking | Upregulated | 0.135512559 | 0.030496259 | 0.131804141 | 2.214923552 | -3.921923445 | 5.665542442 | 5.530029883 |
| SNX27 | Autophagosome-Lysosome Interface | Endosomal sorting (Retromer/SNX) | Upregulated | 0.061254992 | 0.030702612 | 0.131804141 | 2.21207476 | -3.927611725 | 6.464447639 | 6.403192647 |
| CHMP6 | Autophagosome-Lysosome Interface | ESCRT / membrane remodeling | Upregulated | 0.105757917 | 0.032145082 | 0.135578351 | 2.192618154 | -3.966295604 | 3.819526167 | 3.71376825 |
| RAB10 | Autophagosome-Lysosome Interface | Rab/Arl trafficking & effectors | Upregulated | 0.150083388 | 0.03350147 | 0.139229708 | 2.175009912 | -4.001054483 | 7.263045412 | 7.112962024 |
| VPS37A | Autophagosome-Lysosome Interface | Trafficking/sorting (other) | Upregulated | 0.110778301 | 0.035260749 | 0.142722078 | 2.153079501 | -4.044011713 | 6.198355445 | 6.087577145 |
| SNX5 | Autophagosome-Lysosome Interface | Endosomal sorting (Retromer/SNX) | Upregulated | 0.064210117 | 0.037584988 | 0.145982512 | 2.125528822 | -4.097450769 | 6.342306217 | 6.278096101 |
| VPS16 | Autophagosome-Lysosome Interface | Tethering (HOPS/CORVET) | Upregulated | 0.106132478 | 0.041968142 | 0.157844782 | 2.077383669 | -4.189417073 | 4.598754699 | 4.492622221 |
| RAB1A | Autophagosome-Lysosome Interface | Rab/Arl trafficking & effectors | Upregulated | 0.107481773 | 0.043128294 | 0.159387174 | 2.065373624 | -4.212075445 | 7.149070792 | 7.041589019 |
| NSF | Autophagosome-Lysosome Interface | Fusion SNAREs & cofactors | Upregulated | 0.120599042 | 0.046201224 | 0.166402714 | 2.034862636 | -4.269126487 | 5.629210996 | 5.508611954 |
| VPS26C | Autophagosome-Lysosome Interface |  | Upregulated | 0.08643452 | 0.047890348 | 0.171036958 | 2.018830409 | -4.298808974 | 6.066354546 | 5.979920026 |
| TMC8 | Autophagosome-Lysosome Interface | Context-specific | Downregulated | -0.462238974 | 0.000230922 | 0.02890677 | -3.913647662 | 0.424560453 | 4.018500554 | 4.480739528 |
| SLC7A14 | Autophagosome-Lysosome Interface |  | Downregulated | -0.334062488 | 0.002733775 | 0.044686705 | -3.122967375 | -1.815511635 | 1.100517078 | 1.434579566 |
| KIF1A | Autophagosome-Lysosome Interface | Motors/tethers/scaffolds | Downregulated | -0.304228522 | 0.00579314 | 0.066542828 | -2.859579003 | -2.483386267 | 3.223798391 | 3.528026913 |
| GGA2 | Autophagosome-Lysosome Interface | Adaptor complexes & lysosome biogenesis trafficking | Downregulated | -0.121733684 | 0.01154719 | 0.090976457 | -2.604007071 | -3.08858328 | 5.650167895 | 5.771901579 |
| C9orf72 | Autophagosome-Lysosome Interface | Immune/innate regulators linked to autophagy | Downregulated | -0.124513736 | 0.032219796 | 0.135578351 | -2.191631423 | -3.968249716 | 4.626793751 | 4.751307487 |
| TBC1D5 | Autophagosome-Lysosome Interface | Endosomal sorting (Retromer/SNX) | Downregulated | -0.103673873 | 0.029596148 | 0.131804141 | -2.227551939 | -3.89663344 | 6.276284787 | 6.37995866 |
| TBC1D14 | Autophagosome-Lysosome Interface | Trafficking/sorting (other) | Downregulated | -0.082570253 | 0.021099752 | 0.124547144 | -2.367197803 | -3.608966234 | 6.091378395 | 6.173948648 |

Table S5: Transcripts related to Autophagasome-Lysosome Regulatory

| **SYMBOL** | **Primary_Category** | **Subcategory** | **Direction** | **log2FC** | **p_value** | **p_adjusted** | **t** | **B** | **AUD_mean** | **Control_mean** |
| --- | --- | --- | --- | --- | --- | --- | --- | --- | --- | --- |
| LAMTOR5 | Autophagy-Lysosome Regulatory | Nutrient sensing (mTOR/AMPK/Ragulator) | Upregulated | 0.135515192 | 0.000954309 | 0.02890677 | 3.471732408 | -0.867952815 | 4.593203881 | 4.457688688 |
| LAMTOR2 | Autophagy-Lysosome Regulatory | Nutrient sensing (mTOR/AMPK/Ragulator) | Upregulated | 0.167328003 | 0.001092698 | 0.02890677 | 3.427993672 | -0.990497116 | 3.863327353 | 3.695999349 |
| PPARG | Autophagy-Lysosome Regulatory | Metabolic regulators linked to autophagy | Upregulated | 0.288653448 | 0.004984031 | 0.06230039 | 2.913472389 | -2.350268337 | 6.340465244 | 6.051811795 |
| FNBP1L | Autophagy-Lysosome Regulatory | Context-specific | Upregulated | 0.124944061 | 0.005507 | 0.065451871 | 2.87779397 | -2.438603591 | 7.116772796 | 6.991828735 |
| CUL3 | Autophagy-Lysosome Regulatory | Epigenetic/ubiquitin regulation | Upregulated | 0.080144124 | 0.0062103 | 0.069457304 | 2.834459813 | -2.54479115 | 6.493456987 | 6.413312863 |
| DNAJC13 | Autophagy-Lysosome Regulatory | Proteostasis/chaperone network | Upregulated | 0.105503662 | 0.008855924 | 0.081177895 | 2.704077286 | -2.856846068 | 7.087612743 | 6.982109082 |
| MTOR | Autophagy-Lysosome Regulatory | Nutrient sensing (mTOR/AMPK/Ragulator) | Upregulated | 0.097718653 | 0.010816702 | 0.088405738 | 2.628869917 | -3.031648925 | 6.79817193 | 6.700453277 |
| LAMTOR3 | Autophagy-Lysosome Regulatory | Nutrient sensing (mTOR/AMPK/Ragulator) | Upregulated | 0.110008388 | 0.013056932 | 0.091298323 | 2.556854521 | -3.195379161 | 5.283021351 | 5.173012963 |
| LAMTOR1 | Autophagy-Lysosome Regulatory | Nutrient sensing (mTOR/AMPK/Ragulator) | Upregulated | 0.096691392 | 0.013148484 | 0.091298323 | 2.554157006 | -3.201441714 | 4.959733725 | 4.863042333 |
| HSPA1A | Autophagy-Lysosome Regulatory | Proteostasis/chaperone network | Upregulated | 0.289432626 | 0.014875864 | 0.096288072 | 2.506208005 | -3.308348239 | 5.969511693 | 5.680079067 |
| HSPH1 | Autophagy-Lysosome Regulatory | Proteostasis/chaperone network | Upregulated | 0.212816326 | 0.024009513 | 0.12512761 | 2.314509646 | -3.719214892 | 6.167643045 | 5.954826719 |
| PPARGC1A | Autophagy-Lysosome Regulatory | Metabolic regulators linked to autophagy | Upregulated | 0.245886157 | 0.024264125 | 0.12512761 | 2.310174224 | -3.728194852 | 5.511240136 | 5.265353978 |
| BAG1 | Autophagy-Lysosome Regulatory | Proteostasis/chaperone network | Upregulated | 0.132710428 | 0.02739335 | 0.131804141 | 2.259947153 | -3.831204008 | 5.360797316 | 5.228086888 |
| BAG3 | Autophagy-Lysosome Regulatory | Proteostasis/chaperone network | Upregulated | 0.164244173 | 0.030559358 | 0.131804141 | 2.214050649 | -3.923667058 | 4.195315908 | 4.031071735 |
| PRKAA1 | Autophagy-Lysosome Regulatory | Nutrient sensing (mTOR/AMPK/Ragulator) | Upregulated | 0.072313508 | 0.036021504 | 0.144425843 | 2.143891559 | -4.061898618 | 7.340235182 | 7.267921674 |
| ATL3 | Autophagy-Lysosome Regulatory | mTOR regulator | Upregulated | 0.094478509 | 0.037783709 | 0.145982512 | 2.123242839 | -4.101858336 | 6.733146549 | 6.63866804 |
| PLIN3 | Autophagy-Lysosome Regulatory | Metabolic regulators linked to autophagy | Upregulated | 0.213474172 | 0.041503377 | 0.157490491 | 2.082275861 | -4.180154875 | 6.673630918 | 6.460156746 |
| KLF4 | Autophagy-Lysosome Regulatory | Stress-responsive TF/kinase signaling | Upregulated | 0.226079262 | 0.044536243 | 0.161776952 | 2.051168491 | -4.238728442 | 6.622796498 | 6.396717236 |
| STK4 | Autophagy-Lysosome Regulatory | Stress kinase (MST1) that modulates autophagy/apoptosis balance | Downregulated | -0.111959901 | 0.015958573 | 0.099741082 | -2.478659332 | -3.369031679 | 6.400782614 | 6.512742515 |
| ZNF704 | Autophagy-Lysosome Regulatory | Context-specific | Downregulated | -0.17954248 | 0.024418789 | 0.12512761 | -2.307560357 | -3.733602177 | 5.941778696 | 6.121321176 |

Table S6: Clinical metadata and sample information of AUD+ and AUD- subjects

| **Study code** | **Group** | **Site** | **Age** | **Sex** | **Race** | **AUDIT-C Score** |
| --- | --- | --- | --- | --- | --- | --- |
| HVG006 | AUD- | Ileum | 62 | Female | Black | 0 |
| HVG027 | AUD- | Ileum | 51 | Female | Black | 2 |
| HVG029 | AUD- | Ileum | 60 | Female | White | 2 |
| HVG085 | AUD- | Ileum | 61 | Female | White | 1 |
| HAUD015 | AUD- | Ileum | 59 | Female | White | 2 |
| HAUD017 | AUD- | Ileum | 59 | Female | Black | 0 |
| HAUD020 | AUD- | Ileum | 66 | Female | White | 0 |
| MCB319 | AUD- | Ileum | 50 | Female | White | 1 |
| HVG032 | AUD+ | Ileum | 61 | Female | White | 3 |
| HVG070 | AUD+ | Ileum | 49 | Female | White | 4 |
| HVG087 | AUD+ | Ileum | 50 | Female | White | 4 |
| HVG088 | AUD+ | Ileum | 52 | Female | White | 4 |
| HAUD008 | AUD+ | Ileum | 54 | Female | White | 6 |
| MCB282 | AUD+ | Ileum | 46 | Female | White | 7 |
| HVG085 | AUD- | Sigmoid Colon | 61 | Female | White | 1 |
| HAUD015 | AUD- | Sigmoid Colon | 59 | Female | White | 2 |
| HAUD014 | AUD- | Sigmoid Colon | 57 | Female | Black | 2 |
| HAUD017 | AUD- | Sigmoid Colon | 59 | Female | Black | 0 |
| HVG070 | AUD+ | Sigmoid Colon | 49 | Female | White | 4 |
| HVG087 | AUD+ | Sigmoid Colon | 50 | Female | White | 4 |
| HVG088 | AUD+ | Sigmoid Colon | 52 | Female | White | 4 |
| HAUD008 | AUD+ | Sigmoid Colon | 54 | Female | White | 6 |

Table S7: Clinical metadata and sample information of control human colonic organoids

| **Study Code** | **Group** | **Site** | **Age** | **Sex** | **Race** |
| --- | --- | --- | --- | --- | --- |
| WD-109721 | Control | Colon | 55 | Female | Black |
| SD-24575 | Control | Colon | 72 | Male | Black |
| WD-110362 | Control | Colon | 61 | Male | White |

Table S8: PCR primer sequence

| **Gene name** | **Forward Primer** | **Reverse Primer** | **Species** |
| --- | --- | --- | --- |
| *ADH1* | AGTCATCCCACTCGCTATTCC | GTCCCCTGAGGATTGCTTACA | Human |
| *CYP2E1* | GGGAAACAGGGCAATGAGAG | GGAAGGTGGGGTCGAAAGG | Human |
| *ALDH2* | ATGGCAAGCCCTATGTCATCT | CCGTGGTACTTATCAGCCCA | Human |
| *ATG3* | ACACGGTGAAGGGAAAGGC | TGGTGGACTAAGTGATCTCCAG | Human/Mice |
| *ATG4B* | TATGATACTCTCCGGTTTGCTGA | GTTCCCCCAATAGCTGGAAAG | Human/Mice |
| *ATG12* | TGAATCAGTCCTTTGCCCCT | CATGCCTGGGATTTGCAGT | Human/Mice |
| *ATG14* | GAGGGCCTTTACGTGGCTG | AATAGACGAAATCACCGCTCTG | Human/Mice |
| *MAP1LC3B* | TTATAGAGCGATACAAGGGGGAG | CGCCGTCTGATTATCTTGATGAG | Human/ Mice |
| *SQSTM1* | AGGATGGGGACTTGGTTGC | TCACAGATCACATTGGGGTGC | Human/Mice |
| *BECN1* | ATGGAGGGGTCTAAGGCGTC | TCCTCTCCTGAGTTAGCCTCT | Human/Mice |
| *TFEB* | CCAGAAGCGAGAGCTCACAGAT | TGTGATTGTCTTTCTTCTGCCG | Human |
| *LAMP1* | CAGATGTGTTAGTGGCACCCA | TTGGAAAGGTACGCCTGGATG | Human |
| *LAMP2* | GCACAGTGAGCACAAATGAGT | CAGTGGTGTGTATGGTGGGT | Human |
| *LAMP3* | ATGCAGGCAGATTTTAAGTGCT | GTTCTTCGACATGGAAGGGATTT | Human |
| *CTSB* | GAGCTGGTCAACTATGTCAACA | GCTCATGTCCACGTTGTAGAAGT | Human |
| *CTSD* | CACCACAAGTACAACAGCGAC | CCCGAGCCATAGTGGATGT | Human |
| *CTSL* | CTTTTGCCTGGGAATTGCCTC | CATCGCCTTCCACTTGGTC | Human |
| *ACTB* | GTTGTCGACGACGAGCG | GCACAGAGCCTCGCCTT | Human |
| *Atg5* | AGCCAGGTGATGATTCACGG | GGCTGGGGGACAATGCTAA | Mice |
| *Atg7* | GTTCGCCCCCTTTAATAGTGC | TGAACTCCAACGTCAAGCGG | Mice |
| *Atg13* | CCAGGCTCGACTTGGAGAAAA | AGATTTCCACACACATAGATCGC | Mice |
| *Rb1cc1* | GACACTGAGCTAACTGTGCAA | GCGCTGTAAGTACACACTCTTC | Mice |
| *Lamp1* | CAGCACTCTTTGAGGTGAAAAAC | ACGATCTGAGAACCATTCGCA | Mice |
| *Lamp2* | TGTATTTGGCTAATGGCTCAGC | TATGGGCACAAGGAAGTTGTC | Mice |
| *Tfeb* | CCACCCCAGCCATCAACAC | CAGACAGATACTCCCGAACCTT | Mice |
| *Gba1* | CCAAAGACGCATTTTACAAACAG | TCGGGAATGAACTTGCTGAA | Mice |
| *Atp6v0d2* | CAGAGCTGTACTTCAATGTGGAC | AGGTCTCACACTGCACTAGGT | Mice |
| *Atp6v0e1* | CATTGTGATGAGCGTGTTCTGG | AACTCCCCGGTTAGGACCCTTA | Mice |
| *Ctsb* | AGGTGAAGTCCCAGCTTGAAT | GTCTGATAGGAAGACAGGGTCA | Mice |
| *Ctsd* | GCTTCCGGTCTTTGACAACCT | CACCAAGCATTAGTTCTCCTCC | Mice |
| *Ctsl* | ATCAAACCTTTAGTGCAGAGTGG | CTGTATTCCCCGTTGTGTAGC | Mice |
| *Ppargc1a* | ATGTGTCGCCTTCTTGCTCT | ATCTACTGCCTGGGGACCTT | Mice |
| *Vatp6d* | GAGCACAGACTGGTCGAAA | AGCTGTCAGTTCCTTCGTGG | Mice |
| *Vatp6h* | ATGAGTACCGGTTTGCCTGG | GACTGAATGCCAGGAGCCAT | Mice |
| *Cldn2* | GCAAACAGGCTCCGAAGATACT | GAGATGATGCCCAAGTACAGAG | Mice |
| *Tnf* | GCACAGAAAGCATGATCCGC | CCCCATCTTTTGGGGGAGTG | Mice |
| *Il1b* | CACTTCACAAGTCGGAGGCT | CTGCAAGTGCATCATCGTTGT | Mice |
| *Il6* | TCTTTGAAGTTGACGGACCC | TGAGTGATACTGCCTGCCTG | Mice |
| *Il23a* | GCCAAGGTCTGGCTTTTTAT | AGGGAGGTGTGAAGTTGCTC | Mice |
| *Actb* | GCTCCTAGCACCATGAAGAT | GTGTAAAACGCAGCTCAGTA | Mice |
