# Supplementary material for: Alcohol disrupts spatial coordination of autophagy–lysosome pathways to impair intestinal barrier integrity
Source: eBioMedicine. 2026 Oct 26;133:106534. doi: 10.1016/j.ebiom.2026.106534 (PMC13734950; doi:10.1016/j.ebiom.2026.106534)
Supplement: Supplementary file 2 [file mmc2.docx]

**Supplementary Materials and methods:**

**Human RNA-seq data reanalysis**

Bulk RNA-seq data from human duodenal biopsies of individuals with AUD (*n*=44) and control subjects (*n*=16) were reanalyzed from a previously published study^1^. Duodenal biopsies were collected during clinically indicated upper gastrointestinal endoscopy from actively drinking individuals with AUD and from healthy control subjects with normal endoscopic and histological findings, as described in the original publication. Differential gene expression analysis was re-performed using the limma package with empirical Bayes (eBayes) moderation, following the same analytical pipeline as the original study. Reanalyzed data were used for visualization and integration into Fig. 1a-e.

**Ex vivo assessment of intestinal permeability:** Fresh ileal segments were excised from the small intestine at the ileocecal junction and processed as previously described^2^. Segments of comparable length were used for all experiments. One end of each segment was ligated, and 200 μl of FITC-dextran (4 kDa; 40 mg/ml) was gently introduced into the lumen using a gavage needle to prevent mucosal injury. The opposite end was then ligated to generate a sealed intestinal sac. After rinsing in KHBB buffer sacs were incubated in 4 ml KHBB at 37 °C for 20 min. At the end of incubation, FITC-dextran fluorescence in the external buffer was quantified using a fluorometer (excitation 485 nm, emission 520 nm), and permeability values were normalized to the length of each intestinal segment^2^.

**Serum analyses:** Serum alanine aminotransferase (ALT) activity and blood alcohol concentration (BAC) was measured by the Omaha VA Medical Center clinical laboratory using a Vitros automated analyzer (Johnson & Johnson, New Brunswick, NJ). Serum endotoxin levels were quantified using a Limulus amebocyte lysate assay (QCL-1000; Lonza, Walkersville, MD), with *Escherichia coli* endotoxin used to generate the standard curve.

**Hepatic assays:** Hepatic triglyceride content was measured from snap-frozen liver tissue following organic lipid extraction^3^. Briefly, total lipids were extracted, clarified by filtration through Whatman No. 1 filter paper, and dried by vacuum centrifugation. Lipid residues were resuspended in 86.3% (v/v) ethanol containing 0.73 N KOH, and triglycerides were saponified at 65 °C for 20 min. Glycerol content was then quantified spectrophotometrically using the Thermo DMA reagent (Thermo Electron Inc., Middletown, VA) according to the manufacturer’s instructions. Hepatic endotoxin levels were quantified from liver homogenates using a Limulus amebocyte lysate (LAL) assay, as described above.

**Autophagy flux measurement in human organoids**

Colonic organoids derived from AUD+ and AUD− subjects were transduced with the Premo™ Autophagy Tandem Sensor RFP–GFP–LC3B construct (Thermo Fisher Scientific, cat. no. P36239) according to the manufacturer’s instructions. Twenty-four hours after transduction, organoids were washed and media was replaced with fresh ethanol-containing medium (50 mM) for 24 h prior to fixation and imaging.

**Proteasome catalytic assay**

Trypsin- and chymotrypsin-like proteasome activities in mouse intestinal soluble fractions were measured by incubating samples with the fluorogenic substrates Boc-Leu-Arg-Arg-AMC (UBPBio, cat. no. G3100) and Suc-Leu-Leu-Val-Tyr-AMC (UBPBio, cat. no. G1101), respectively. Release of AMC fluorescence was quantified as previously described^4^.

**RNA extraction and real-time PCR**

Total RNA was extracted from organoids using a Qiagen RNeasy Mini Kit (cat. no. 74034) and from mouse whole tissues using TRIzol reagent. cDNA was synthesized using a qScript cDNA Synthesis Kit (Quanta Bio, cat. no. 95047-500). mRNA expression was quantified by SYBR Green–based quantitative real-time PCR (qRT-PCR) using gene-specific primers and SYBR Green qPCR SuperMix (Quanta Bio, cat. no. 95072-012). Primer sequences are listed in Table S8. Relative mRNA levels were normalized to Actb (β-actin).

**Protein extraction, fractionation, and immunoblot analysis**

Total protein lysates were prepared from mouse whole tissue and isolated crypts of duodenal and ileal samples using RIPA buffer supplemented with protease and phosphatase inhibitor cocktail (Thermo Fisher Scientific, cat. no. 1861281), as previously described^5^. Soluble and insoluble protein fractions were prepared from mucosal scrapings as described previously^6^. Briefly, mucosal scrapings were incubated for 15 min in lysis buffer containing 50 mM Tris (pH 7.4), 150 mM NaCl, 2 mM EDTA, 1.0% Triton X-100, and protease and phosphatase inhibitor cocktail. Lysates were then centrifuged at 15,600 × g for 4 min at 4 °C, and the supernatant was collected as the detergent-soluble fraction. The remaining pellet was sonicated in 100–200 μl of lysis buffer containing 10 mM Tris (pH 7.4), 1.0% SDS, and protease and phosphatase inhibitor cocktail to obtain the detergent-insoluble fraction. Protein concentrations were determined using a BCA assay. Equal amounts of protein were resolved on 7.5% or 12% Mini-PROTEAN TGX precast gels (Bio-Rad, cat. no. 4561093) and transferred to nitrocellulose membranes (Bio-Rad, cat. no. 1620112). Membranes were blocked in 5% non-fat dry milk and incubated overnight at 4 °C with primary antibodies against mTOR (RRID: AB_330978, cat. no. 2972, Cell Signaling Technology; 1:250 dilution), phospho-mTOR (RRID: AB_330970, cat. no. 2971, Cell Signaling Technology; 1:250 dilution), LC3B (RRID: AB_10003146, cat. no. NB100-2220, Novus Biologics; 1:500 dilution), ATG7 (RRID: AB_10831194, cat. no. 8558, Cell Signaling Technology; 1:500, dilution), ATG12 (RRID: AB_1903898, cat. no. 4180, Cell Signaling Technology; 1:500 dilution), ATG16L1 (RRID: AB_10950320, cat. no. 8089, Cell Signaling Technology; 1:500 dilution), Beclin-1 (RRID: AB_2259061, cat. no. 11306-1-AP, Proteintech; 1:500 dilution), p62 (SQSTM1) (RRID: AB_437085, cat. no. H00008878-M01, Abnova; 1:500 dilution), cathepsin B (RRID: AB_2687580, cat. no. 31718S, Cell Signaling Technology; 1:500 dilution), LAMP1 (RRID: AB_2134495, cat. no. SC-19992, Santa Cruz Biotechnology; 1:250 dilution), CHOP (RRID: AB_2089254, cat. no. 2895, Cell Signaling Technology; 1:1000 dilution), PDI (RRID: AB_2298935, cat. no. 2446, Cell Signaling Technology; 1:1000 dilution), elF2α (RRID: AB_2230924, cat. no. 9722S, Cell Signaling Technology; 1:1000 dilution), phospho-elF2α (RRID: AB_390740, cat. no. 3597S, Cell Signaling Technology; 1:500 dilution), STAT3 (RRID: AB_331269, cat. no. 4904S, Cell Signaling Technology; 1:500 dilution), phospho-STAT3 (RRID: AB_2491009, cat. no. 9145S, Cell Signaling Technology; 1:500 dilution) and claudin-2 (RRID: AB_2533085, cat. no. 32-5600, Invitrogen; 1:1000 dilution), β-Actin (RRID: AB_476744, cat. no. A5441. Sigma-Aldrich; 1:1000 dilution), GAPDH (RRID: AB_561053, cat. no. 2118L, Cell Signaling Technology; 1:1000 dilution). Membranes were washed and incubated with infrared dye–conjugated secondary antibodies (LI-COR Biosciences; goat anti-rat, cat. no. 926-32219; goat anti-rabbit, cat. no. 926-68071; goat anti-mouse, cat. no. 926-32210; 1:20,000 dilution) for 1 h at room temperature. Immunoreactive bands were visualized using the LI-COR Odyssey DLx imaging system and quantified by densitometry using FIJI (ImageJ). Protein loading was normalized to GAPDH or β-actin, as indicated.

**Immunohistochemistry (IHC), immunofluorescence (IF), and imaging**

**Human tissue**: FFPE sections were deparaffinized and rehydrated through graded ethanol series (5 min per step). Sections were permeabilized, blocked, and incubated overnight at 4 °C with primary antibodies against LC3B (Novus Biologicals; 1:500 dilution), p62 (RRID: AB_2687579, Abcam, cat.no. ab56416; 1:500 dilution), LAMP1 (RRID: AB_2134495, cat.no. 9091s, Cell Signaling Technology; 1:500 dilution), TFEB (RRID: AB_2316009, cat. no. 6801, ProSci; 1:500, dilution), and claudin-2 (cat. no. 32-5600, Invitrogen; 1:200). For IHC, after washing to remove unbound primary antibody, sections were incubated with HRP-conjugated secondary antibodies (Diagnostic Biosystems, cat. no K055; 1:500 dilution) and developed using a chromogenic substrate. Where nuclear localization and quantification of nuclear protein staining were required, slides were counterstained with hematoxylin. All IHC sections were imaged using a Nikon brightfield microscope equipped with a DS-10 color camera. For IF, sections were washed and incubated with Alexa Fluor–conjugated secondary antibodies (goat anti-mouse 488 (cat. no. A32723, **RRID:** [AB_2633275](https://www.antibodyregistry.org/AB_2633275FAFF4)), donkey anti-rabbit 594 (cat. no. A32795, RRID: AB_2762835), or chicken anti-rat 647 (cat. no. A-21470, RRID: AB_2535875); 1:500 dilution, Invitrogen). Nuclei were counterstained with DAPI using VECTASHIELD® mounting medium (Vector Laboratories). Images were acquired using Nikon confocal microscopy platforms controlled by NIS-Elements software. Immunofluorescence imaging of human and mouse tissue sections was performed using a Nikon Crest Cicero spinning-disk confocal microscope, whereas human intestinal organoids were imaged using a Nikon AXR confocal microscope. Z-stack images were acquired using identical laser power, detector gain, and exposure settings within each experiment. Image stacks were denoised, deconvolved, and converted into maximum-intensity projections (MIP) using identical processing parameters across groups prior to analysis.

**Human intestinal organoids**: Organoids were fixed in 4% paraformaldehyde in PBS for 15 min, washed three times in PBS, and permeabilized with 0.3% Triton X-100 for 5 min. Following blocking, organoids were incubated overnight at 4 °C with primary antibodies against LC3B, p62 (SQSTM1), LAMP1, and claudin-2 (antibody details provided in the Immunoblotting and Human IF/IHC sections). Organoids were then incubated with species-appropriate Alexa Fluor–conjugated secondary antibodies as described above. Nuclei were counterstained with DAPI, and images were acquired by confocal microscopy and processed as described above.

**Mouse tissue**: Mouse duodenal and ileal tissues embedded in OCT compound (Tissue-Tek) at the time of collection were cryosectioned at 5–8 µm and processed for immunofluorescence staining. Sections were incubated overnight at 4 °C with primary antibodies against LC3B, p62 (SQSTM1), LAMP1, and claudin-2 (antibody details provided in the Immunoblotting and Human IF/IHC sections), followed by incubation with Alexa Fluor–conjugated secondary antibodies as described above. Nuclei were counterstained with DAPI, and images were acquired using confocal microscopy and processed as described above.

**Image analysis**: All image analyses were performed using FIJI (NIH ImageJ). For tissue immunohistochemistry (IHC) and immunofluorescence (IF) analyses, regions of interest (ROIs) corresponding to crypt and villus compartments were manually delineated for each section, whereas entire organoids were analyzed for human organoid studies. Staining intensity was quantified following background subtraction using identical thresholding parameters across groups. LC3B-, p62-, and LAMP1-positive puncta were quantified by applying uniform thresholding and binarization, followed by morphological opening to remove background noise and isolate discrete puncta. Puncta number and size were quantified using the Analyze Particles function in FIJI and normalized to the number of nuclei per field. Manders’ overlap coefficients (MOC) were calculated on thresholded images using the Coloc2 plugin. Manders’ overlap coefficients were calculated using both single optical sections and maximum-intensity projections (MIP), yielding comparable results. Puncta quantification from single optical planes likewise did not differ significantly from measurements obtained from MIP images.

**References**

1.	Llorente C, Raya Tonetti F, Bruellman R, et al. mAChR4 suppresses liver disease via GAP-induced antimicrobial immunity. Nature 2025;646:180-189.

2.	Kirpich IA, Feng W, Wang Y, et al. The type of dietary fat modulates intestinal tight junction integrity, gut permeability, and hepatic toll-like receptor expression in a mouse model of alcoholic liver disease. Alcohol Clin Exp Res 2012;36:835-46.

3.	Thomes PG, Rasineni K, Yang L, et al. Ethanol withdrawal mitigates fatty liver by normalizing lipid catabolism. Am J Physiol Gastrointest Liver Physiol 2019;316:G509-G518.

4.	Thomes PG, Strupp MS, Donohue TM, Jr., et al. Hydroxysteroid 17beta-dehydrogenase 11 accumulation on lipid droplets promotes ethanol-induced cellular steatosis. J Biol Chem 2023;299:103071.

5.	Gyongyosi B, Cho Y, Lowe P, et al. Alcohol-induced IL-17A production in Paneth cells amplifies endoplasmic reticulum stress, apoptosis, and inflammasome-IL-18 activation in the proximal small intestine in mice. Mucosal Immunol 2019;12:930-944.

6.	Rao RK, Basuroy S, Rao VU, et al. Tyrosine phosphorylation and dissociation of occludin-ZO-1 and E-cadherin-beta-catenin complexes from the cytoskeleton by oxidative stress. Biochem J 2002;368:471-81.
