# Supplementary material for: Alcohol disrupts spatial coordination of autophagy–lysosome pathways to impair intestinal barrier integrity
Source: eBioMedicine. 2026 Oct 26;133:106534. doi: 10.1016/j.ebiom.2026.106534 (PMC13734950; doi:10.1016/j.ebiom.2026.106534)
Supplement: Supplementary file 3 [file mmc3.docx]

**Supplementary Figures**

**Alcohol disrupts spatial coordination of autophagy-lysosome pathways across the intestinal epithelium**

Murugadas Anbazhagan^1^, Ahmed Bakheet^1^, John D Dickinson^2^, Ali Keshavarzian^3^, Nghi V. N. Tran^4^, Tin Nguyen^5,6^, Paul G Thomes^1*^

**Contents**

**Supplementary Figure S1.** Additional quantification of TFEB staining intensity and nuclear–cytoplasmic distribution in ileal epithelium from individuals with AUD (AUD+) and without AUD (AUD−).

**Supplementary Figure S2.** mRNA expression of ethanol metabolism-, autophagy-, and lysosome-related transcripts in human colonic organoids derived from individuals with AUD (AUD+) and without AUD (AUD−).

**Supplementary Figure S3.** Tandem LC3 reporter–based autophagy flux analysis in human colonic organoids from individuals with AUD (AUD+) and without AUD (AUD−) under control and ethanol-exposed conditions.

**Supplementary Figure S4.** Additional quantification of LC3B, p62, and LAMP1 colocalization in human colonic organoids exposed to ethanol.

**Supplementary Figure S5.** Basal and apical epithelial localization of p62, LC3B, and LAMP1 and puncta size quantification in duodenal villus and crypt compartments of control- and ethanol-fed mice.

**Supplementary Figure S6.** Additional representative images and puncta size quantification of LC3B, p62, and LAMP1 in ileal crypt and villus epithelium from control- and ethanol-fed mice.

**Supplementary Figure S7.** Representative images of claudin-2 association with autophagy–lysosome compartments in human and mouse intestinal crypts.

**Supplementary Figure S1:**

**a)	b)	c)**

**Supplementary Figure S2:**

**a)	b)	c)**

**Supplementary Figure S3:**

**b)	c)	d)**

**e)	f)	g)**

**h)	i)	j)**

**k)	l)	m)**

**Supplementary Figure S4:**

**a)	b)	c)**

**Supplementary Figure S5:**

**c)	d)	e)**

**Supplementary Figure S6:**

**c)	d)	e)**

**Supplementary Figure S7:**

**Human Ileal Crypts**

**a)	Claudin2	LC3B	Claudin2/ LC3B	b) Claudin2	LAMP1	Claudin2/ LAMP1**

**AUD-**

**AUD+**

**c)	d)	e)**

**Mouse Duodenal Crypts**

**f)	Claudin2	LC3B	Claudin2/ LC3B	g) Claudin2	LAMP1	Claudin2/ LAMP1**

**Ethanol+**

**Control-**

**h)	i)	j)**

**Mouse Ileal Crypts**

**k) Claudin2	LC3B	Claudin2/ LC3B	l)	Claudin2	LAMP1	Claudin2/ LAMP1**

**Control-**

**Ethanol+**

**m)	n)	0)**

**Figure legends.**

**Supplementary Figure S1:** **Immunohistochemical (IHC) quantification of TFEB staining intensity in human ileal epithelium**. Quantification of nuclear, cytoplasmic, and nuclear/cytoplasmic TFEB staining intensities, and nuclear–cytoplasmic TFEB ratio, in crypt and villus compartments of human ileal epithelium from individuals with AUD (AUD+) and without AUD (AUD−) (*n* = 3 donors per group).

**Supplementary Figure S2:** **Baseline mRNA expression of ethanol metabolism, autophagy, and lysosome pathways in human colonic organoids**. mRNA expression levels of (a) ethanol metabolism–related, (b) autophagy-related, and (c) lysosome-related transcripts in colonic organoids derived from individuals with AUD (AUD+) and without AUD (AUD−) (*n* = 3 independent donor-derived organoid lines per group).

**Supplementary Figure S3:** **Tandem GFP–RFP–LC3 reporter analysis in human colonic organoids derived from** individuals with AUD and individuals without AUD**.** (a) Representative immunofluorescence images of human colonic organoids derived from individuals with AUD (AUD+) and individuals without AUD (AUD−) transduced with tandem GFP–RFP–LC3 reporter and exposed to 0 or 50 mM ethanol for 24 h. Quantification of GFP⁺RFP⁻ (green), GFP⁺RFP⁺ (yellow), and GFP⁻RFP⁺ (red) puncta is shown for (b–d) AUD− organoids ± ethanol, (e–g) AUD+ organoids ± ethanol, and (h–j) AUD+ vs AUD− organoids. (k–m) Red fraction was calculated as red/(red + yellow) to quantify the relative enrichment of red-only puncta within the RFP-positive LC3 pool and to enable comparison across conditions with differing total puncta counts in the treatment groups shown in (b–j). Quantification was performed using five organoids per donor from three independent donor-derived organoid lines per category (total *n* = 15 organoids per group), with each organoid containing approximately 500 cells (~7,500 cells per group). Statistical comparisons between two groups were performed using two-tailed, unpaired student’s *t*-tests.

**Supplementary Figure S4:** **LC3B, p62, and LAMP1 colocalisation in ethanol-exposed human colonic organoids.** Manders’ colocalisation analysis for LC3B, p62, and LAMP1 in human colonic organoids exposed to 50 mM ethanol for 24 h (related to Fig. 1j). (a) LC3B–p62, (b) p62–LAMP1, and (c) LC3B–LAMP1 colocalization. Quantification was performed using three organoids per donor, with ~500 cells per organoid (~4,500 cells per group). Statistical comparisons between two groups were performed using two-tailed, unpaired student’s *t*-tests

**Supplementary Figure S5: LC3B, p62, and LAMP1 epithelial localisation in duodenal villi and puncta size in villus and crypt compartments of ethanol-fed mice.** (a, b) Representative immunofluorescence images of duodenal villus and crypt compartments, respectively, from control- and ethanol diet–fed mice stained for LC3B (green), p62 (red), and LAMP1 (magenta). In villus epithelium, a white arrowhead indicates p62 signal enriched towards the basal region, whereas a white arrow indicates LC3B and LAMP1 enriched towards the apical region. (c) LC3B puncta size quantification, (d) p62 puncta size quantification, and (e) LAMP1 puncta size quantification (*n* = 3 animals per group). For puncta size quantification, a minimum of 5 crypts and 3 villi per mouse were quantified across 3 mice per group, yielding a total of 15 crypts and 9 villi per group. Statistical comparisons between two groups were performed using two-tailed, unpaired student’s *t*-tests.

**Supplementary Figure S6: LC3B, p62, and LAMP1 puncta size in ileal villus and crypt compartments of ethanol-fed mice.** (a, b) Representative immunofluorescence images of ileal villus and crypt compartments, respectively, from control- and ethanol diet–fed mice stained for LC3B (green), p62 (red), and LAMP1 (magenta). In villus epithelium, a white arrowhead indicates p62 signal enriched toward the basal region, whereas a white arrow indicates LC3B and LAMP1 enriched towards the apical region. (c–e) Quantification of LC3B puncta size, p62 puncta size, and LAMP1 puncta size in ileal villus and crypt compartments from control- and ethanol diet–fed mice (*n* = 3 animals per group). For puncta size quantification, a minimum of 5 crypts and 3 villi per mouse were quantified across 3 mice per group, yielding a total of 15 crypts and 9 villi per group. Statistical comparisons between two groups were performed using two-tailed, unpaired student’s *t*-tests.

**Supplementary Figure S7: Ethanol exposure alters claudin-2 association with autophagy–lysosome compartments in human and mouse intestinal crypts.**

Representative immunofluorescence micrographs depicting colocalisation of claudin-2 with LC3B and LAMP1, together with corresponding quantification of staining intensity and Manders’ colocalisation coefficients, are shown for each experimental condition as indicated. **(a–e)** Ileal crypts from individuals with AUD (AUD+) and individuals without AUD (AUD−) (*n* = 3 per group). **(f-j)** Duodenal crypts from control- and ethanol-fed mice (*n* = 3 animals per group). **(k-o)** Ileal crypts from control- and ethanol-fed mice (*n* = 3 animals per group). For human ileal tissue, 3–6 crypts per donor were quantified across three independent donors, yielding 9–18 crypts per group. For mouse studies, a minimum of 3-4 crypts per mouse were quantified across four mice per group (total 9-12 crypts per group). Statistical comparisons between two groups were performed using two-tailed, unpaired student’s *t*-tests.
